# Supplementary material for: The impacts of hydropower on freshwater macroinvertebrate richness: A global meta-analysis
Source: PLoS One. 2022 Aug 18;17(8):e0273089. doi: 10.1371/journal.pone.0273089 (PMC9387867; doi:10.1371/journal.pone.0273089)
Supplement: S1 Fig — No statistically significant asymmetry is observed (z = -1.74, P = 0.08). (DOCX) [file pone.0273089.s001.docx]

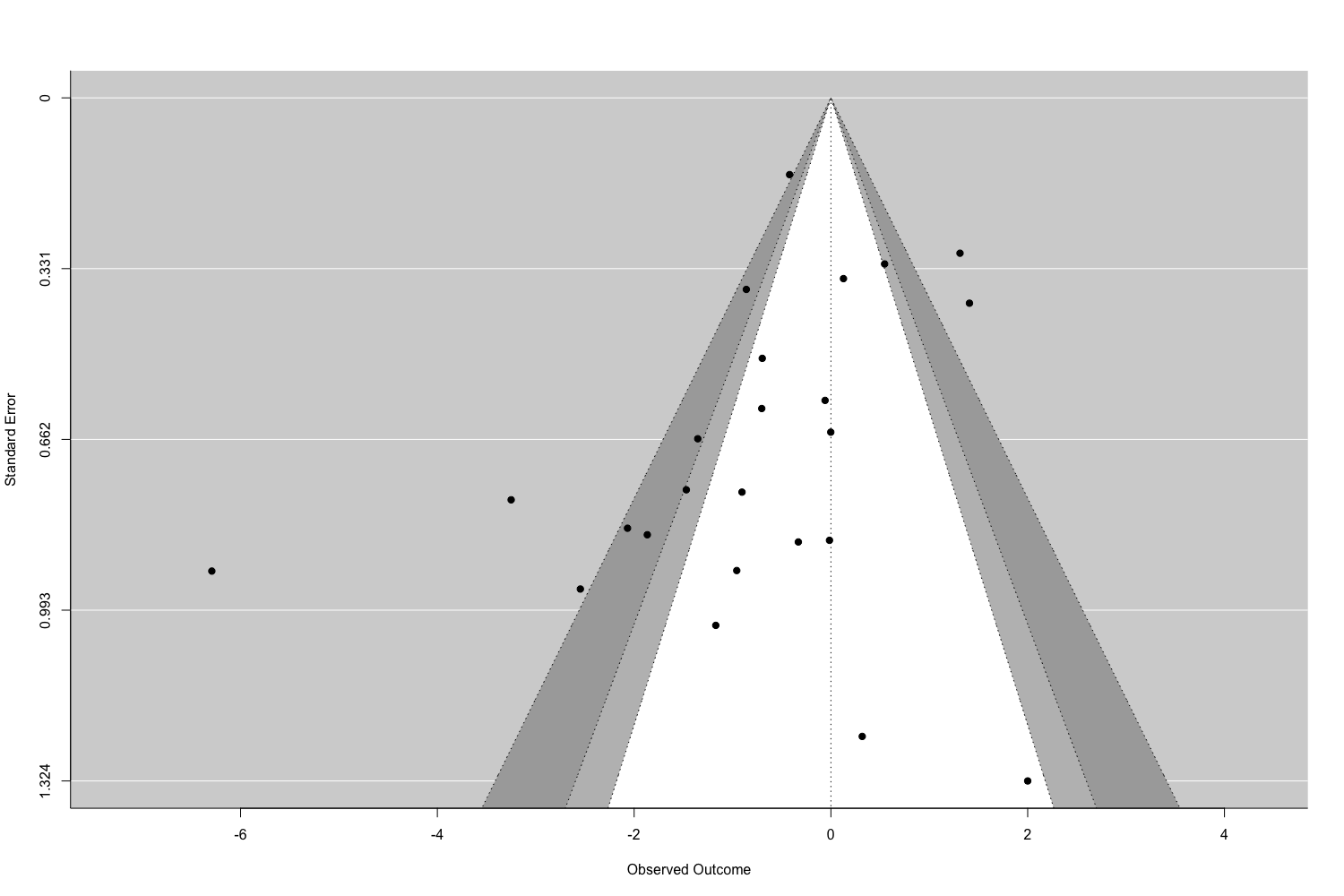


**S1 Fig.** **Funnel plot for this meta-analysis.** No statistically significant asymmetry is observed (z = -1.74, P = 0.08).
