# Supplementary material for: The impacts of hydropower on freshwater macroinvertebrate richness: A global meta-analysis
Source: PLoS One. 2022 Aug 18;17(8):e0273089. doi: 10.1371/journal.pone.0273089 (PMC9387867; doi:10.1371/journal.pone.0273089)
Supplement: S2 Table — Use S3 Table as a companion table to get more information on each variable. (DOCX) [file pone.0273089.s003.docx]

**S2 Table.** **Metadata table showing all variables for each studies included in this meta-analysis.** Use S3 Table as a companion table to get more information on each variable.

| **UID.M** | **UID.FP** | **AUTHORS** | **YEAR** | **OBS** | **BIOME** | **IMP** | **STUDY** | **SEASON** | **GEAR** | **REF.MEAN** | **REF.SD** | **REF.N** | **IMP.MEAN** | **IMP.SD** | **IMP.N** | **YI** | **VI** | **WI** | **CI.LOW** | **CI.UP** |
| --- | --- | --- | --- | --- | --- | --- | --- | --- | --- | --- | --- | --- | --- | --- | --- | --- | --- | --- | --- | --- |
| 1 | 56 | Aroviita and Hamalainen | 2008 | 3 | BOR | WLF | NAT.IMP | FALL | GRAB | 18,80 | 5,39 | 11 | 14,17 | 5,74 | 6 | -0,80 | 0,28 | 0,08 | -1,07 | -0,52 |
| 1 | 63 | Aroviita and Hamalainen | 2008 | 1 | BOR | WLF | NAT.IMP | FALL | NET | 35,83 | 4,22 | 6 | 27,80 | 8,64 | 5 | -1,12 | 0,42 | 0,08 | -1,54 | -0,69 |
| 1 | 73 | Aroviita and Hamalainen | 2008 | 4 | BOR | WLF | NAT.IMP | FALL | GRAB | 18,80 | 5,39 | 11 | 10,80 | 1,92 | 5 | -1,62 | 0,37 | 0,08 | -1,99 | -1,25 |
| 1 | 84 | Aroviita and Hamalainen | 2008 | 2 | BOR | WLF | NAT.IMP | FALL | NET | 35,83 | 4,22 | 6 | 24,20 | 3,90 | 5 | -2,60 | 0,67 | 0,08 | -3,28 | -1,93 |
| 2 | 104 | Valdovinos et al. | 2007 | 2 | TEM | WLF | NAT.IMP | SUMMER | GRAB | 16,70 | 3,50 | 16 | 2,75 | 0,50 | 16 | -5,44 | 0,59 | 0,14 | -6,03 | -4,85 |
| 2 | 107 | Valdovinos et al. | 2007 | 1 | TEM | WLF | NAT.IMP | SUMMER | GRAB | 19,00 | 3,00 | 16 | 1,50 | 0,60 | 16 | -7,89 | 1,10 | 0,14 | -8,98 | -6,79 |
| 3 | 2 | Marchetti et al. | 2011 | 3 | TEM | FR | GRAD | SUMMER | NET | 19,50 | 0,30 | 4 | 21,00 | 0,40 | 4 | 3,69 | 1,35 | 0,05 | 2,34 | 5,03 |
| 3 | 16 | Marchetti et al. | 2011 | 6 | TEM | FR | GRAD | SUMMER | NET | 16,60 | 2,10 | 4 | 20,10 | 2,60 | 4 | 1,29 | 0,60 | 0,05 | 0,68 | 1,89 |
| 3 | 22 | Marchetti et al. | 2011 | 4 | TEM | FR | GRAD | WINTER | NET | 13,60 | 1,60 | 4 | 15,00 | 1,60 | 4 | 0,76 | 0,54 | 0,05 | 0,22 | 1,30 |
| 3 | 54 | Marchetti et al. | 2011 | 1 | TEM | FR | GRAD | WINTER | NET | 17,50 | 0,70 | 4 | 16,50 | 1,50 | 4 | -0,74 | 0,53 | 0,05 | -1,28 | -0,21 |
| 3 | 71 | Marchetti et al. | 2011 | 2 | TEM | FR | GRAD | SPRING | NET | 20,00 | 0,70 | 4 | 19,00 | 0,40 | 4 | -1,52 | 0,65 | 0,05 | -2,17 | -0,88 |
| 3 | 77 | Marchetti et al. | 2011 | 5 | TEM | FR | GRAD | SPRING | NET | 18,40 | 1,30 | 4 | 14,80 | 1,80 | 4 | -1,99 | 0,75 | 0,05 | -2,74 | -1,24 |
| 4 | 4 | Molozzi et al. | 2013 | 2 | TRO | WLF | NAT.IMP | NA | GRAB | 20,50 | 2,12 | 2 | 29,00 | 0,00 | 2 | 3,20 | 2,28 | 0,11 | 0,92 | 5,48 |
| 4 | 15 | Molozzi et al. | 2013 | 1 | TRO | WLF | NAT.IMP | NA | GRAB | 20,50 | 2,12 | 2 | 27,50 | 3,54 | 2 | 1,35 | 1,23 | 0,11 | 0,12 | 2,58 |
| 5 | 75 | Takao et al. | 2008 | 1 | TEM | FR | NAT.IMP | WINTER | NET | 79,88 | 8,50 | 4 | 62,74 | 7,42 | 4 | -1,87 | 0,72 | 0,29 | -2,58 | -1,15 |
| 6 | 31 | Kullasoot et al. | 2017 | 2 | TRO | FR | GRAD | NA | NET | 10,33 | 4,92 | 6 | 12,83 | 5,86 | 6 | 0,43 | 0,34 | 0,05 | 0,09 | 0,77 |
| 6 | 32 | Kullasoot et al. | 2017 | 7 | TRO | FR | GRAD | NA | NET | 10,33 | 4,92 | 6 | 13,00 | 7,01 | 6 | 0,41 | 0,34 | 0,05 | 0,07 | 0,75 |
| 6 | 35 | Kullasoot et al. | 2017 | 4 | TRO | FR | GRAD | NA | NET | 10,33 | 4,92 | 6 | 11,61 | 3,77 | 6 | 0,27 | 0,34 | 0,05 | -0,07 | 0,61 |
| 6 | 43 | Kullasoot et al. | 2017 | 5 | TRO | FR | GRAD | NA | NET | 10,33 | 4,92 | 6 | 10,17 | 5,96 | 6 | -0,03 | 0,33 | 0,05 | -0,36 | 0,31 |
| 6 | 53 | Kullasoot et al. | 2017 | 3 | TRO | FR | GRAD | NA | NET | 10,33 | 4,92 | 6 | 6,06 | 6,11 | 6 | -0,71 | 0,35 | 0,05 | -1,06 | -0,36 |
| 6 | 55 | Kullasoot et al. | 2017 | 6 | TRO | FR | GRAD | NA | NET | 10,33 | 4,92 | 6 | 6,78 | 3,19 | 6 | -0,79 | 0,36 | 0,05 | -1,15 | -0,43 |
| 7 | 33 | White et al. | 2011 | 1 | BOR | WLF | NAT.IMP | SUMMER | NET | 24,86 | 4,38 | 14 | 26,88 | 5,72 | 8 | 0,40 | 0,20 | 0,11 | 0,20 | 0,60 |
| 7 | 52 | White et al. | 2011 | 2 | BOR | WLF | NAT.IMP | SUMMER | NET | 24,86 | 4,38 | 14 | 21,44 | 5,35 | 8 | -0,69 | 0,21 | 0,11 | -0,90 | -0,49 |
| 7 | 85 | White et al. | 2011 | 3 | BOR | WLF | NAT.IMP | SUMMER | NET | 24,86 | 4,38 | 14 | 13,88 | 3,04 | 8 | -2,67 | 0,36 | 0,11 | -3,02 | -2,31 |
| 8 | 1 | Smokorowski et al. | 2011 | 1 | TEM | FR | NAT.IMP | SUMMER | CB | 14,00 | 0,73 | 30 | 18,00 | 0,77 | 30 | 5,26 | 0,30 | 0,12 | 4,96 | 5,56 |
| 8 | 8 | Smokorowski et al. | 2011 | 3 | TEM | FR | NAT.IMP | SUMMER | CB | 15,00 | 0,90 | 30 | 17,00 | 0,80 | 30 | 2,32 | 0,11 | 0,12 | 2,21 | 2,43 |
| 8 | 40 | Smokorowski et al. | 2011 | 2 | TEM | FR | NAT.IMP | SUMMER | CB | 17,00 | 0,78 | 30 | 17,00 | 0,78 | 30 | 0,00 | 0,07 | 0,12 | -0,07 | 0,07 |
| 9 | 51 | Englund and Malmqvist | 1996 | 1 | BOR | FR | NAT.IMP | NA | NET | 80,00 | 4,46 | 14 | 74,00 | 11,20 | 16 | -0,67 | 0,14 | 0,17 | -0,81 | -0,53 |
| 9 | 61 | Englund and Malmqvist | 1996 | 2 | BOR | FR | NAT.IMP | NA | NET | 80,00 | 4,46 | 14 | 71,00 | 10,22 | 21 | -1,04 | 0,13 | 0,17 | -1,18 | -0,91 |

**S2 Table.** (continued)

| **UID.M** | **UID.FP** | **AUTHORS** | **YEAR** | **OBS** | **BIOME** | **IMP** | **STUDY** | **SEASON** | **GEAR** | **REF.MEAN** | **REF.SD** | **REF.N** | **IMP.MEAN** | **IMP.SD** | **IMP.N** | **YI** | **VI** | **WI** | **CI.LOW** | **CI.UP** |
| --- | --- | --- | --- | --- | --- | --- | --- | --- | --- | --- | --- | --- | --- | --- | --- | --- | --- | --- | --- | --- |
| 10 | 25 | Jackson et al. | 2007 | 9 | TEM | FR | GRAD | WINTER | CB | 14,77 | 3,16 | 10 | 17,42 | 4,06 | 5 | 0,72 | 0,32 | 0,03 | 0,40 | 1,04 |
| 10 | 50 | Jackson et al. | 2007 | 6 | TEM | FR | GRAD | FALL | CB | 23,19 | 3,02 | 10 | 21,00 | 3,68 | 5 | -0,64 | 0,31 | 0,03 | -0,95 | -0,32 |
| 10 | 64 | Jackson et al. | 2007 | 3 | TEM | FR | GRAD | SPRING | CB | 19,12 | 2,98 | 10 | 14,97 | 4,35 | 5 | -1,13 | 0,34 | 0,03 | -1,47 | -0,79 |
| 10 | 66 | Jackson et al. | 2007 | 7 | TEM | FR | GRAD | WINTER | CB | 14,77 | 3,16 | 10 | 10,20 | 3,85 | 5 | -1,27 | 0,35 | 0,03 | -1,62 | -0,92 |
| 10 | 69 | Jackson et al. | 2007 | 8 | TEM | FR | GRAD | WINTER | CB | 14,77 | 3,16 | 10 | 9,91 | 3,58 | 5 | -1,39 | 0,36 | 0,03 | -1,75 | -1,02 |
| 10 | 88 | Jackson et al. | 2007 | 5 | TEM | FR | GRAD | FALL | CB | 23,19 | 3,02 | 10 | 13,13 | 4,38 | 5 | -2,71 | 0,54 | 0,03 | -3,25 | -2,16 |
| 10 | 95 | Jackson et al. | 2007 | 2 | TEM | FR | GRAD | SPRING | CB | 19,12 | 2,98 | 10 | 9,03 | 1,62 | 5 | -3,60 | 0,73 | 0,03 | -4,33 | -2,87 |
| 10 | 101 | Jackson et al. | 2007 | 4 | TEM | FR | GRAD | FALL | CB | 23,19 | 3,02 | 10 | 10,32 | 2,37 | 5 | -4,27 | 0,91 | 0,03 | -5,18 | -3,36 |
| 10 | 106 | Jackson et al. | 2007 | 1 | TEM | FR | GRAD | SPRING | CB | 19,12 | 2,98 | 10 | 3,24 | 1,92 | 5 | -5,54 | 1,32 | 0,03 | -6,86 | -4,22 |
| 11 | 24 | Kraft | 1988 | 4 | TEM | WLF | NAT.IMP | SUMMER | GRAB | 7,90 | 1,60 | 5 | 9,10 | 1,30 | 5 | 0,74 | 0,43 | 0,05 | 0,32 | 1,17 |
| 11 | 27 | Kraft | 1988 | 5 | TEM | WLF | NAT.IMP | SUMMER | GRAB | 8,70 | 2,60 | 5 | 10,30 | 2,20 | 5 | 0,60 | 0,42 | 0,05 | 0,18 | 1,02 |
| 11 | 30 | Kraft | 1988 | 3 | TEM | WLF | NAT.IMP | SUMMER | GRAB | 5,80 | 2,00 | 5 | 6,90 | 1,90 | 5 | 0,51 | 0,41 | 0,05 | 0,10 | 0,92 |
| 11 | 46 | Kraft | 1988 | 2 | TEM | WLF | NAT.IMP | SUMMER | GRAB | 5,30 | 1,60 | 5 | 4,60 | 0,90 | 5 | -0,49 | 0,41 | 0,05 | -0,90 | -0,07 |
| 11 | 47 | Kraft | 1988 | 6 | TEM | WLF | NAT.IMP | SUMMER | GRAB | 8,20 | 2,70 | 5 | 6,90 | 1,50 | 5 | -0,54 | 0,41 | 0,05 | -0,95 | -0,12 |
| 11 | 57 | Kraft | 1988 | 1 | TEM | WLF | NAT.IMP | SUMMER | GRAB | 5,50 | 1,70 | 5 | 4,30 | 0,60 | 5 | -0,85 | 0,44 | 0,05 | -1,29 | -0,41 |
| 12 | 3 | Mellado-Diaz et al. | 2019 | 9 | TEM | FR | GRAD | NA | NET | 49,69 | 1,75 | 9 | 55,37 | 0,69 | 3 | 3,23 | 0,93 | 0,02 | 2,30 | 4,16 |
| 12 | 5 | Mellado-Diaz et al. | 2019 | 14 | TEM | FR | GRAD | NA | NET | 41,64 | 4,07 | 9 | 52,34 | 2,90 | 3 | 2,55 | 0,72 | 0,02 | 1,84 | 3,27 |
| 12 | 23 | Mellado-Diaz et al. | 2019 | 17 | TEM | FR | GRAD | NA | NET | 39,02 | 1,45 | 7 | 40,30 | 1,78 | 2 | 0,75 | 0,60 | 0,02 | 0,15 | 1,36 |
| 12 | 38 | Mellado-Diaz et al. | 2019 | 13 | TEM | FR | GRAD | NA | NET | 41,64 | 4,07 | 9 | 42,35 | 3,82 | 3 | 0,16 | 0,45 | 0,02 | -0,28 | 0,61 |
| 12 | 49 | Mellado-Diaz et al. | 2019 | 5 | TEM | FR | GRAD | NA | NET | 46,53 | 2,59 | 4 | 44,37 | 3,79 | 3 | -0,58 | 0,68 | 0,02 | -1,26 | 0,09 |
| 12 | 60 | Mellado-Diaz et al. | 2019 | 7 | TEM | FR | GRAD | NA | NET | 46,53 | 2,59 | 4 | 44,02 | 1,01 | 3 | -0,94 | 0,72 | 0,02 | -1,66 | -0,22 |
| 12 | 78 | Mellado-Diaz et al. | 2019 | 10 | TEM | FR | GRAD | NA | NET | 49,69 | 1,75 | 9 | 45,49 | 2,51 | 3 | -2,03 | 0,66 | 0,02 | -2,69 | -1,37 |
| 12 | 79 | Mellado-Diaz et al. | 2019 | 12 | TEM | FR | GRAD | NA | NET | 41,64 | 4,07 | 9 | 32,98 | 3,04 | 3 | -2,06 | 0,62 | 0,02 | -2,68 | -1,44 |
| 12 | 81 | Mellado-Diaz et al. | 2019 | 4 | TEM | FR | GRAD | NA | NET | 43,22 | 2,28 | 4 | 34,27 | 4,20 | 3 | -2,36 | 0,98 | 0,02 | -3,34 | -1,38 |
| 12 | 83 | Mellado-Diaz et al. | 2019 | 1 | TEM | FR | GRAD | NA | NET | 43,22 | 2,28 | 4 | 33,67 | 4,39 | 3 | -2,44 | 1,01 | 0,02 | -3,45 | -1,43 |
| 12 | 87 | Mellado-Diaz et al. | 2019 | 6 | TEM | FR | GRAD | NA | NET | 46,53 | 2,59 | 4 | 39,53 | 0,54 | 3 | -2,69 | 1,21 | 0,02 | -3,90 | -1,48 |
| 12 | 90 | Mellado-Diaz et al. | 2019 | 16 | TEM | FR | GRAD | NA | NET | 39,02 | 1,45 | 7 | 34,48 | 0,50 | 2 | -3,05 | 1,07 | 0,02 | -4,12 | -1,98 |
| 12 | 92 | Mellado-Diaz et al. | 2019 | 15 | TEM | FR | GRAD | NA | NET | 39,02 | 1,45 | 7 | 32,96 | 2,05 | 2 | -3,44 | 1,20 | 0,02 | -4,64 | -2,23 |

**S2 Table.** (continued)

| **UID.M** | **UID.FP** | **AUTHORS** | **YEAR** | **OBS** | **BIOME** | **IMP** | **STUDY** | **SEASON** | **GEAR** | **REF.MEAN** | **REF.SD** | **REF.N** | **IMP.MEAN** | **IMP.SD** | **IMP.N** | **YI** | **VI** | **WI** | **CI.LOW** | **CI.UP** |
| --- | --- | --- | --- | --- | --- | --- | --- | --- | --- | --- | --- | --- | --- | --- | --- | --- | --- | --- | --- | --- |
| 12 | 97 | Mellado-Diaz et al. | 2019 | 8 | TEM | FR | GRAD | NA | NET | 46,53 | 2,59 | 4 | 35,01 | 2,29 | 3 | -3,80 | 1,76 | 0,02 | -5,55 | -2,04 |
| 12 | 98 | Mellado-Diaz et al. | 2019 | 2 | TEM | FR | GRAD | NA | NET | 43,22 | 2,28 | 4 | 31,32 | 2,73 | 3 | -4,05 | 1,76 | 0,02 | -5,81 | -2,30 |
| 12 | 100 | Mellado-Diaz et al. | 2019 | 11 | TEM | FR | GRAD | NA | NET | 49,69 | 1,75 | 9 | 41,99 | 1,59 | 3 | -4,11 | 1,21 | 0,02 | -5,32 | -2,90 |
| 12 | 102 | Mellado-Diaz et al. | 2019 | 3 | TEM | FR | GRAD | NA | NET | 43,22 | 2,28 | 4 | 28,62 | 2,95 | 3 | -4,78 | 2,21 | 0,02 | -6,99 | -2,56 |
| 13 | 37 | Bruno et al. | 2019 | 1 | TEM | FR | NAT.IMP | NA | NET | 35,60 | 1,90 | 5 | 36,20 | 3,80 | 10 | 0,17 | 0,30 | 0,16 | -0,13 | 0,47 |
| 13 | 76 | Bruno et al. | 2019 | 2 | TEM | FR | NAT.IMP | NA | NET | 38,60 | 5,10 | 5 | 28,40 | 4,90 | 10 | -1,93 | 0,42 | 0,16 | -2,36 | -1,51 |
| 14 | 21 | Milner et al. | 2019 | 2 | TEM | FR | NAT.IMP | SUMMER | NET | 31,30 | 10,69 | 3 | 40,33 | 7,77 | 3 | 0,77 | 0,72 | 0,10 | 0,05 | 1,49 |
| 14 | 48 | Milner et al. | 2019 | 3 | TEM | FR | NAT.IMP | SUMMER | NET | 31,33 | 2,31 | 3 | 28,33 | 5,86 | 3 | -0,54 | 0,69 | 0,10 | -1,23 | 0,15 |
| 14 | 68 | Milner et al. | 2019 | 1 | TEM | FR | NAT.IMP | SUMMER | NET | 31,00 | 1,00 | 3 | 19,67 | 9,45 | 3 | -1,35 | 0,82 | 0,10 | -2,16 | -0,53 |
| 15 | 45 | Steel et al. | 2018 | 1 | TEM | FR | NAT.IMP | SUMMER | NET | 36,67 | 3,06 | 3 | 35,00 | 2,65 | 3 | -0,47 | 0,68 | 0,09 | -1,15 | 0,22 |
| 15 | 62 | Steel et al. | 2018 | 2 | TEM | FR | NAT.IMP | SUMMER | NET | 31,33 | 9,45 | 3 | 21,33 | 4,16 | 3 | -1,09 | 0,77 | 0,09 | -1,86 | -0,33 |
| 15 | 72 | Steel et al. | 2018 | 3 | TEM | FR | NAT.IMP | SUMMER | NET | 36,67 | 3,06 | 3 | 25,00 | 8,49 | 2 | -1,53 | 1,07 | 0,09 | -2,60 | -0,47 |
| 16 | 29 | Schneider and Petrin | 2017 | 1 | BOR | FR | NAT.IMP | SUMMER | NET | 10,82 | 3,89 | 20 | 14,01 | 7,11 | 20 | 0,55 | 0,10 | 0,35 | 0,44 | 0,65 |
| 17 | 67 | Vaikasas et al. | 2013 | 2 | TEM | FR | GRAD | SPRING | NET | 28,00 | 1,73 | 3 | 20,00 | 6,32 | 10 | -1,29 | 0,50 | 0,15 | -1,79 | -0,79 |
| 17 | 93 | Vaikasas et al. | 2013 | 1 | TEM | FR | GRAD | SPRING | NET | 28,00 | 1,73 | 3 | 17,00 | 3,16 | 10 | -3,47 | 0,90 | 0,15 | -4,36 | -2,57 |
| 18 | 36 | Doledec et al. | 2021 | 1 | TEM | FR | GRAD | NA | NET | 22,50 | 7,64 | 144 | 24,00 | 7,00 | 72 | 0,20 | 0,02 | 0,09 | 0,18 | 0,22 |
| 18 | 39 | Doledec et al. | 2021 | 2 | TEM | FR | GRAD | NA | NET | 22,50 | 7,64 | 144 | 23,00 | 5,00 | 72 | 0,07 | 0,02 | 0,09 | 0,05 | 0,09 |
| 18 | 59 | Doledec et al. | 2021 | 3 | TEM | FR | GRAD | NA | NET | 22,50 | 7,64 | 144 | 16,00 | 6,00 | 72 | -0,91 | 0,02 | 0,09 | -0,93 | -0,88 |
| 18 | 65 | Doledec et al. | 2021 | 4 | TEM | FR | GRAD | NA | NET | 22,50 | 7,64 | 144 | 14,00 | 6,00 | 72 | -1,19 | 0,02 | 0,09 | -1,21 | -1,16 |
| 19 | 70 | Wang et al. | 2016 | 6 | TEM | FR | GRAD | SUMMER | NET | 16,57 | 2,36 | 9 | 12,90 | 2,60 | 3 | -1,41 | 0,53 | 0,05 | -1,93 | -0,88 |
| 19 | 74 | Wang et al. | 2016 | 4 | TEM | FR | GRAD | SUMMER | NET | 16,57 | 2,36 | 9 | 12,40 | 0,87 | 3 | -1,79 | 0,58 | 0,05 | -2,37 | -1,21 |
| 19 | 86 | Wang et al. | 2016 | 3 | TEM | FR | GRAD | SUMMER | NET | 16,57 | 2,36 | 9 | 9,00 | 3,46 | 3 | -2,67 | 0,74 | 0,05 | -3,41 | -1,93 |
| 19 | 89 | Wang et al. | 2016 | 5 | TEM | FR | GRAD | SUMMER | NET | 16,57 | 2,36 | 9 | 10,30 | 0,69 | 3 | -2,71 | 0,75 | 0,05 | -3,46 | -1,96 |
| 19 | 99 | Wang et al. | 2016 | 2 | TEM | FR | GRAD | SUMMER | NET | 16,57 | 2,36 | 9 | 6,60 | 1,73 | 3 | -4,09 | 1,14 | 0,05 | -5,23 | -2,95 |
| 19 | 105 | Wang et al. | 2016 | 1 | TEM | FR | GRAD | SUMMER | NET | 16,57 | 2,36 | 9 | 3,70 | 1,04 | 3 | -5,49 | 1,70 | 0,05 | -7,20 | -3,79 |
| 20 | 6 | Nukazawa et al. | 2020 | 6 | TEM | FR | GRAD | SUMMER | NET | 28,70 | 4,00 | 18 | 46,00 | 8,10 | 30 | 2,47 | 0,15 | 0,04 | 2,32 | 2,63 |
| 20 | 9 | Nukazawa et al. | 2020 | 5 | TEM | FR | GRAD | WINTER | NET | 32,00 | 3,50 | 30 | 47,70 | 8,70 | 54 | 2,13 | 0,08 | 0,04 | 2,05 | 2,21 |

**S2 Table.** (continued)

| **UID.M** | **UID.FP** | **AUTHORS** | **YEAR** | **OBS** | **BIOME** | **IMP** | **STUDY** | **SEASON** | **GEAR** | **REF.MEAN** | **REF.SD** | **REF.N** | **IMP.MEAN** | **IMP.SD** | **IMP.N** | **YI** | **VI** | **WI** | **CI.LOW** | **CI.UP** |
| --- | --- | --- | --- | --- | --- | --- | --- | --- | --- | --- | --- | --- | --- | --- | --- | --- | --- | --- | --- | --- |
| 20 | 11 | Nukazawa et al. | 2020 | 8 | TEM | FR | GRAD | SUMMER | NET | 28,70 | 4,00 | 18 | 50,30 | 12,60 | 30 | 2,06 | 0,13 | 0,04 | 1,93 | 2,20 |
| 20 | 13 | Nukazawa et al. | 2020 | 1 | TEM | FR | GRAD | WINTER | NET | 32,00 | 3,50 | 30 | 44,01 | 7,70 | 54 | 1,82 | 0,07 | 0,04 | 1,75 | 1,89 |
| 20 | 14 | Nukazawa et al. | 2020 | 3 | TEM | FR | GRAD | WINTER | NET | 32,00 | 3,50 | 30 | 47,40 | 13,00 | 54 | 1,43 | 0,06 | 0,04 | 1,37 | 1,50 |
| 20 | 17 | Nukazawa et al. | 2020 | 10 | TEM | FR | GRAD | SUMMER | NET | 28,70 | 4,00 | 18 | 34,60 | 5,50 | 30 | 1,16 | 0,10 | 0,04 | 1,06 | 1,26 |
| 20 | 18 | Nukazawa et al. | 2020 | 7 | TEM | FR | GRAD | SUMMER | NET | 28,70 | 4,00 | 18 | 37,40 | 9,90 | 30 | 1,04 | 0,10 | 0,04 | 0,94 | 1,14 |
| 20 | 20 | Nukazawa et al. | 2020 | 2 | TEM | FR | GRAD | WINTER | NET | 32,00 | 3,50 | 30 | 39,20 | 10,20 | 54 | 0,84 | 0,06 | 0,04 | 0,79 | 0,90 |
| 20 | 26 | Nukazawa et al. | 2020 | 9 | TEM | FR | GRAD | SUMMER | NET | 28,70 | 4,00 | 18 | 31,50 | 4,10 | 30 | 0,68 | 0,09 | 0,04 | 0,58 | 0,77 |
| 20 | 28 | Nukazawa et al. | 2020 | 4 | TEM | FR | GRAD | WINTER | NET | 32,00 | 3,50 | 30 | 37,30 | 10,70 | 54 | 0,59 | 0,05 | 0,04 | 0,54 | 0,65 |
| 21 | 34 | Quadroni | 2020 | 2 | TEM | FR | NAT.IMP | SUMMER | NET | 19,04 | 6,27 | 15 | 20,47 | 3,47 | 18 | 0,28 | 0,12 | 0,17 | 0,16 | 0,41 |
| 21 | 42 | Quadroni | 2020 | 1 | TEM | FR | NAT.IMP | SUMMER | NET | 15,15 | 3,00 | 15 | 15,04 | 4,99 | 18 | -0,03 | 0,12 | 0,17 | -0,15 | 0,10 |
| 22 | 58 | Vilenica | 2020 | 1 | TEM | WLF | NAT.IMP | SUMMER | NET | 6,00 | 1,00 | 3 | 2,80 | 3,70 | 5 | -0,90 | 0,58 | 0,30 | -1,49 | -0,32 |
| 23 | 82 | Uieda and Marcal | 2022 | 3 | TRO | WLF | GRAD | SUMMER | CB | 12,57 | 1,21 | 9 | 10,12 | 0,69 | 9 | -2,37 | 0,38 | 0,07 | -2,75 | -1,99 |
| 23 | 91 | Uieda and Marcal | 2021 | 2 | TRO | WLF | GRAD | SUMMER | CB | 12,57 | 1,21 | 9 | 9,43 | 0,57 | 9 | -3,16 | 0,50 | 0,07 | -3,66 | -2,66 |
| 23 | 94 | Uieda and Marcal | 2023 | 4 | TRO | WLF | GRAD | SUMMER | CB | 12,57 | 1,21 | 9 | 9,12 | 0,52 | 9 | -3,53 | 0,57 | 0,07 | -4,10 | -2,96 |
| 23 | 103 | Uieda and Marcal | 2020 | 1 | TRO | WLF | GRAD | SUMMER | CB | 12,57 | 1,21 | 9 | 7,55 | 0,45 | 9 | -5,24 | 0,98 | 0,07 | -6,22 | -4,25 |
| 24 | 7 | Cazaubon and Giudicelli | 2006 | 8 | TEM | FR | GRAD | FALL | NET | 18,00 | 1,41 | 2 | 24,00 | 1,41 | 2 | 2,40 | 1,72 | 0,03 | 0,68 | 4,12 |
| 24 | 10 | Cazaubon and Giudicelli | 2003 | 5 | TEM | FR | GRAD | SUMMER | NET | 10,50 | 2,12 | 2 | 18,50 | 2,12 | 2 | 2,13 | 1,57 | 0,03 | 0,56 | 3,70 |
| 24 | 12 | Cazaubon and Giudicelli | 2005 | 7 | TEM | FR | GRAD | FALL | NET | 18,00 | 1,41 | 2 | 23,00 | 1,41 | 2 | 2,00 | 1,50 | 0,03 | 0,50 | 3,50 |
| 24 | 19 | Cazaubon and Giudicelli | 2004 | 6 | TEM | FR | GRAD | SUMMER | NET | 10,50 | 2,12 | 2 | 17,50 | 4,95 | 2 | 1,04 | 1,13 | 0,03 | -0,10 | 2,17 |
| 24 | 41 | Cazaubon and Giudicelli | 2002 | 4 | TEM | FR | GRAD | SPRING | NET | 26,50 | 0,71 | 2 | 26,50 | 3,54 | 2 | 0,00 | 1,00 | 0,03 | -1,00 | 1,00 |
| 24 | 44 | Cazaubon and Giudicelli | 2001 | 3 | TEM | FR | GRAD | SPRING | NET | 26,50 | 0,71 | 2 | 24,50 | 3,54 | 2 | -0,44 | 1,02 | 0,03 | -1,47 | 0,58 |
| 24 | 80 | Cazaubon and Giudicelli | 2000 | 2 | TEM | FR | GRAD | WINTER | NET | 24,00 | 0,00 | 2 | 12,00 | 4,24 | 2 | -2,26 | 1,64 | 0,03 | -3,90 | -0,62 |
| 24 | 96 | Cazaubon and Giudicelli | 1999 | 1 | TEM | FR | GRAD | WINTER | NET | 24,00 | 0,00 | 2 | 11,00 | 2,83 | 2 | -3,67 | 2,68 | 0,03 | -6,34 | -0,99 |
