# Supplementary material for: The impacts of hydropower on freshwater macroinvertebrate richness: A global meta-analysis
Source: PLoS One. 2022 Aug 18;17(8):e0273089. doi: 10.1371/journal.pone.0273089 (PMC9387867; doi:10.1371/journal.pone.0273089)
Supplement: S3 Table — (DOCX) [file pone.0273089.s004.docx]

**S3 Table.** **Companion table describing all variables in Table S2.**

| **VARIABLES** | **DESCRIPTION** | **TYPE** |
| --- | --- | --- |
| UID.M | Study/paper unique identifier, relative to the map | Numerical |
| UID.FP | Study/paper unique identifier, relative to the forest plot | Numerical |
| AUTHORS | List of authors, written as if they were cited in a paper | Character string |
| YEAR | Publication year | Numerical |
| OBS | Identification number for each observation in the study | Numerical |
| BIOME | Biomes derived from terrestrial ecoregions as defined by the World Wildlife Fund (WWF; boreal [BOR], temperate [TEM] and tropical [TRO]) | Categorical |
| IMP | Type of impact from hydropower, either flow regulation [FLOW.REG] or water level fluctuation [WLF] | Categorical |
| STUDY | Type of study, either longitudinal gradient [GRAD] or cross-sectional (natural vs impacted [NAT.IMP]) | Categorical |
| SEASON | Season samples were collected [SPRING, SUMMER, FALL, WINTER]. Samples that were collected over multiple seasons (composite) are referred to as NA | Categorical |
| GEAR | Sampling gear used to sample macroinvertebrates; d-nets, kick-sample and Surber [NET], Ponar and Ekman [GRAB] and colonization baskets [COL.BASKET] | Categorical |
| REF.MEAN | Mean richness in reference sample (number of taxa) | Numerical |
| REF.SD | Standard deviation of mean richness in reference sample | Numerical |
| REF.N | Sample number for mean richness in reference sample | Numerical |
| IMP.MEAN | Mean richness in impacted sample (number of taxa) | Numerical |
| IMP.SD | Standard deviation of mean richness in impacted sample | Numerical |
| IMP.N | Sample number for mean richness in impacted sample | Numerical |
| YI | Observed effect sizes (*g*) | Numerical |
| VI | Sampling variance corresponding to respective effect sizes (*V_g_*) | Numerical |
| WI | Inverse variance as weight associated to each study | Numerical |
| CI.LOW | Lower confidence interval | Numerical |
| CI.UP | Upper confidence interval | Numerical |
| NOTES | Description of impact the effect size represents | Character string |
