# Supplementary material for: The impacts of hydropower on freshwater macroinvertebrate richness: A global meta-analysis
Source: PLoS One. 2022 Aug 18;17(8):e0273089. doi: 10.1371/journal.pone.0273089 (PMC9387867; doi:10.1371/journal.pone.0273089)
Supplement: S4 Table — Rho values (ρ) ranges from 0 to 1 and mean effect size (ES), standard error (SE) and between study variance (τ2) estimates are relatively insensitive to these varying ρ values. (DOCX) [file pone.0273089.s005.docx]

**S4 Table.** **Table showing the sensitive analysis outputs.** Rho values ($\rho$) ranges from 0 to 1 and mean effect size (ES), standard error (SE) and between study variance ($\tau^{2}$) estimates are relatively insensitive to these varying $\rho$ values.

| **Estimate type** | $\boldsymbol{\rho}$ **= 0** | $\boldsymbol{\rho}$ **= 0.2** | $\boldsymbol{\rho}$ **= 0.4** | $\boldsymbol{\rho}$ **= 0.6** | $\boldsymbol{\rho}$ **= 0.8** | $\boldsymbol{\rho}$ **= 1** |
| --- | --- | --- | --- | --- | --- | --- |
| Mean effect size (ES) | -0.84 | -0.84 | -0.84 | -0.85 | -0.84 | -0.84 |
| Standard error (SE) | 0.38 | 0. 38 | 0. 38 | 0. 38 | 0. 38 | 0. 38 |
| Between-study variance ($\tau^{2}$) | 2.73 | 2.73 | 2.74 | 2.74 | 2.74 | 2.74 |
