# Supplementary material for: The impacts of hydropower on freshwater macroinvertebrate richness: A global meta-analysis
Source: PLoS One. 2022 Aug 18;17(8):e0273089. doi: 10.1371/journal.pone.0273089 (PMC9387867; doi:10.1371/journal.pone.0273089)
Supplement: Attachment — Submitted filename: PONE-D-22-07760_R1jrs.pdf [file pone.0273089.s007.pdf]

PLOS ONE

The impacts of hydropower on freshwater macroinvertebrate richness: A global meta-analysis

--Manuscript Draft--

**Manuscript Number:**	PONE-D-22-07760R1
**Article Type:**	Research Article
**Full Title:**	The impacts of hydropower on freshwater macroinvertebrate richness: A global meta-analysis
**Short Title:**	The impacts of hydropower on freshwater macroinvertebrate richness
**Corresponding Author:**	Gabrielle Trottier, PhD<br>CIRAIG - Polytechnique Montreal<br>Montréal, Quebec CANADA
**Keywords:**	Meta-analysis; Effect size; robust variance estimation; Heterogeneity; PRISMA; Macroinvertebrates; Hydropower; Reservoirs; dams; moderators; biomes; study designs; impact types; sampling seasons; sampling gears; richness; biodiversity
**Abstract:**	Hydroelectric dams and their reservoirs have been suggested to affect freshwater biodiversity. However, studies investigating the consequences of hydroelectric dams and reservoirs on macroinvertebrate richness have reached opposite conclusions. We carried out a meta-analysis devised to elucidate the effects of hydropower, dams and reservoirs on macroinvertebrate richness while accounting for the potential role played by moderators such as biomes, impact types, study designs, sampling seasons and gears. We used a random/mixed-effects model, combined with robust variance estimation, to conduct the meta-analysis on 107 pairs of observations (i.e. , impacted versus reference) extracted from 24 studies (more than one observation per study). Hydropower, dams and reservoirs did significantly impact macroinvertebrate richness in a clear, directional and statistically significant way, where macroinvertebrate richness in hydropower, dams and reservoirs impacted environments were significantly lower than in unimpacted environments. However, we also observed a large range of effect sizes, from very negative to very positive impacts of hydropower. We tried to account for the large variability in effect sizes using moderators, but none of the moderators included in the meta-analysis had statistically significant effects. This suggests that some other moderators (unavailable for the 24 studies) might be important (e.g. , temperature, granulometry, wave disturbance and macrophytes) and that macroinvertebrate richness may be driven by local, smaller scale processes. As new studies become available, it would be interesting to keep enriching this meta-analysis, as well as collecting local habitat variables, to see if we could statistically strengthen and deepen the conclusions of this meta-analysis.
**Order of Authors:**	Gabrielle Trottier, PhD
	Katrine Turgeon, PhD
	Daniel Boisclair, PhD
	Cécile Bulle, PhD
	Manuele Margni, PhD
**Opposed Reviewers:**	
**Response to Reviewers:**	Reviewers' comments:<br><br>Reviewer's Responses to Questions<br><br>Comments to the Author<br><br>1. Is the manuscript technically sound, and do the data support the conclusions?<br><br>The manuscript must describe a technically sound piece of scientific research with data that supports the conclusions. Experiments must have been conducted rigorously, with

Powered by Editorial Manager® and ProduXion Manager® from Aries Systems Corporation

appropriate controls, replication, and sample sizes. The conclusions must be drawn appropriately based on the data presented.

Reviewer #1: Yes

Reviewer #2: Yes

2. Has the statistical analysis been performed appropriately and rigorously?

Reviewer #1: Yes

Reviewer #2: Yes

3. Have the authors made all data underlying the findings in their manuscript fully available?

The PLOS Data policy requires authors to make all data underlying the findings described in their manuscript fully available without restriction, with rare exception (please refer to the Data Availability Statement in the manuscript PDF file). The data should be provided as part of the manuscript or its supporting information, or deposited to a public repository. For example, in addition to summary statistics, the data points behind means, medians and variance measures should be available. If there are restrictions on publicly sharing data—e.g. participant privacy or use of data from a third party—those must be specified.

Reviewer #1: Yes

Reviewer #2: Yes

4. Is the manuscript presented in an intelligible fashion and written in standard English?

PLOS ONE does not copyedit accepted manuscripts, so the language in submitted articles must be clear, correct, and unambiguous. Any typographical or grammatical errors should be corrected at revision, so please note any specific errors here.

Reviewer #1: Yes

Reviewer #2: Yes

5. Review Comments to the Author

Please use the space provided to explain your answers to the questions above. You may also include additional comments for the author, including concerns about dual publication, research ethics, or publication ethics. (Please upload your review as an attachment if it exceeds 20,000 characters)

REVIEWER #1

Review of Trottier et al.
The authors of this manuscript, “The impacts of hydropower on freshwater macroinvertebrate richness: a global meta-analysis” assess the impacts of hydropower dams on macroinvertebrate richness. They conduct a meta-analysis of the literature, and attempt to account for several potential moderators, including the biome the studies occurred in; whether the study sites occurred upstream (within the reservoir) or downstream of the dam; the study design; the sampling season; and the type of sampling gear used. Using a random and mixed effect modelling approach, the authors conclude that hydropower does significantly impact macroinvertebrate richness, but they observed a large range of effect sizes that were not accounted for by the included moderators, and suggest including local habitat variables in future studies would be helpful in understanding the response of macroinvertebrate richness to hydropower impacts.

Overall, I thought the meta-analysis was well laid out and clear (following the PRISMA

approach), however, there were a few areas of the manuscript that were confusing and require clarification. The authors focus their meta-analysis specifically on macroinvertebrate richness. In their introduction, they mention three aspects of biodiversity: richness, community assemblage and functional diversity. It might be helpful to provide some additional rationale as to why the decision to focus specifically on richness. Is richness more easily quantifiable and extractable for a meta-analyses? While functional diversity measures are likely more limited in the literature, community composition data is frequently assessed, and might also be insightful in looking at hydropower impacts. This might be beyond the scope of this paper, but perhaps some additional rationale to focusing on richness may be helpful.

*** Response: As it was pointed out by the reviewer, we focused this meta-analysis on macroinvertebrate richness because it was more easily quantifiable/extractable in this context. It took a lot of effort to access simple taxon richness data for 24 studies. As community composition and functional diversity are more data hungry and a lot more limited in term of accessibility and computability than taxon richness, they were not as easy to retrieve at the meta-analysis' scale. We added a statement at the end of the introduction (lines 134-137), as well as a sentence saying it would be interesting to conduct further studies using these biodiversity aspects in the conclusion (lines 601-604).

The authors included sampling gear as a proxy for habitat stratum (i.e. benthic grabs vs nets). This approach might need a caveat, as many biomonitoring protocols (including the EPA in the US) use nets for multiple-habitat assessments, whereas grabs are primarily used in deeper reservoir studies (upstream).

*** Response: We thank the reviewer for bringing this issue to our attention. We added a caveat inspired by the reviewer's suggestion (lines 115-119).

The authors included a comprehensive literature search using the Web of Science core collection, and provide the terms used. In the research strategy (line 208; Figure1), the authors state that they reduced the number of included papers to 24, but in the data collection section (Line 220) mention collecting metrics from 17 studies. There does not seem to be any mention on how the number of studies was reduced from 24 (Table S2 does list 24 studies).

*** Response: This was a typographical error. It was corrected to 24 studies.

The seasonal sampling moderator (spring/summer/fall/winter) seems very coarse. For either upstream draw down or downstream modifications in stream flow, were samples collected immediately after alterations or later in the season? As some macroinvertebrates are relatively mobile, they could recolonize shortly after short-term water regulation effects.

*** Response: This is true. However, since this is a meta-analysis, it was not easy to choose an appropriate coarseness for this specific moderator, especially considering some of the studies were not very specific about their sampling. We could have used Julian day, but we were more interested in the coarser temporal scale effect of seasons rather than micro changes in colonization following immediate water regulation. We have added justification to this choice in the manuscript (lines 131-133).

In the data collection/extraction section, it might be helpful to include information on whether multiple authors were involved in abstracting the data, or if this was done by a single analyst.

*** Response: We added this information to the data collection paragraph (lines 220-221).

For samples from reservoirs, it might be useful to know whether samples were collected in the center of the lake or in the littoral regions. The authors state (Line 518) they were expecting a large effect of water level fluctuations. If these were samples collected from the profundal region in the center of the lake, water level drawdown might not have a large impact.

Powered by Editorial Manager® and ProduXion Manager® from Aries Systems Corporation

*** Response: After verification, all samples from reservoir studies were collected in the littoral zone, not in the profundal zone. We specified types of samples at line 518.

Minor edits:

Line 477: The authors state “despite the documented effects on macroinvertebrate richness.” while these are discussed briefly elsewhere in the manuscript, It would be helpful to include some citations with this statement.

*** Response: We added appropriate references for this statement.

Line 496: states that the authors did not have a statistical supporting trend for both boreal and tropical, but previously (lines 395-397), they state they did make statistically valid inferences for both temperate and tropical biomes (just not for boreal).

*** Response: We could indeed make valid statistical inferences, but for both temperate and tropical biome, the confidence interval encompassed zero, so we can say with confidence that there is no statistically significant effect of tropical and temperate biomes, whereas for the boreal biome, we cannot even interpret with confidence the statistical trend observed. We added clarifications to minimize reader confusion (lines 395-398)

Overall, I thought the meta-analysis was interesting, perhaps it might be worth mentioning that in the discussing/conclusion that additional analyses of community composition and functional diversity would also be an important future direction in assessing the impacts of hydropower on macroinvertebrate communities. As mentioned by the authors in their introduction, taxon richness is only one aspect of biodiversity.

*** Response: We thank the reviewer for its valuable feedback. We added a sentence mentioning the future relevance of additional community composition and functional diversity analyses (lines 601-604).

REVIEWER #2

This paper describes a meta-analysis of the literature asking whether hydropower, dams, and reservoirs affect macroinvertebrate species richness. The authors found that a preponderance of studies found negative effects on species richness. However, there was a range of effect sizes in their dataset and despite having a number of likely moderators that might explain variation between studies, the authors found that these moderators were not effective in explaining effect size variation.

Generally this study was well conceived and completed. I liked the detailed description of how the meta-analysis was done and that they used the PRISMA methodology for conducing a meta-analysis.

One thing I’d like to see clarified is what is being asked of the moderators. Are you asking if the moderators impact species richness, or if the moderators explain heterogeneity in the effect sizes? I think it is the latter, but this could be made more clear. I suppose that’s why you call them moderators and not covariates?

*** Response: We are indeed asking the moderators to explain additional heterogeneity, as suggested by the reviewer. We added a sentence to clarify this matter in the manuscript (lines 312-314).

Although I felt there was a lack of detail explaining this aspect of the study, there are at times places where it felt like there was too much detail (see specific comments below).

In addition, a careful screening of the paper would be useful to address various grammatical mistakes – I’ve highlighted some below – which will make the paper more easily assessable to the reader.

Minor comments:

Powered by Editorial Manager® and ProduXion Manager® from Aries Systems Corporation

Line 30 and throughout (e.g., 292, 302, 303, 305): mixed effects models are mixed because they contain fixed and random effects. It is therefore redundant to say "random and mixed effect model".

*** Response: We thank the reviewer for bringing this matter to our attention. We reviewed this formulation and felt comfortable using "random/mixed-effects model" (as it is used in Viechtbauer [2010]) instead of "random and mixed effect model" throughout the manuscript.

Line 33-34: change "statistical way" to "statistically significant way".

*** Response: We changed it according to the reviewer suggestion.

Line 55: add comma before "but"

*** Response: We added a comma.

Line 72: between and "natural-like" flows?

*** Response: We added "impacted flows" (it became missing during the back and forth between co-authors revision of the manuscript pre-submission).

Line 74: delete "allow to"

*** Response: We thank the reviewer for taking the time to highlight these grammatical/syntax errors, we deleted "allow to".

Line 83-84: Why would this latitudinal effect on species richness affect effect sizes? Perhaps more likely to see effects in tropics than higher latitudes because a floor effect makes it difficult to detect change in richness? In any case, it would help the reader to explain the reasoning rather than leaving the reader to connect the dots.

*** Response: We are not entirely sure what the reviewer meant by "floor effect". Nonetheless, we were able to clarify our reasoning and added few lines at two locations in the manuscript (lines 85-87 and 488-496) stating that the loss of one taxon over few taxa is relatively more important than over multiple taxa and thus, why we could potentially observe an effect of a latitudinal gradient on our effect size (i.e., species richness highest in the tropics and lowest near the poles).

Lines 99-104: Most of these study designs are not in your dataset – or even likely as you point out. What isn't mentioned here are the two study designs you did use in your models.

*** Response: The study designs found in our meta-analysis are in facts two variants of the Control-Impact sampling design (line 128-131), that is cross-sectional (i.e., reference natural lake versus impacted reservoir) and longitudinal spatial gradient (i.e., upstream of a dam [reservoir] versus downstream of a dam [river]). In lines 99-104, we simply make our case that sampling, or study design could affect the conclusions of a study.

Lines 137: A little overly wordy – does meta-analysis really need this much defense as a method for the audience of PLoS One?

*** Response: We deleted this paragraph. We agree meta-analysis does not need as much defense and description in PLOS ONE paper.

Line 183: Why this date (1989)?

*** Response: This is the earliest year you can search for articles in Web of Science, we specified it in the manuscript.

Line 198: Why was sorting required? If you went through every result, then sorting is irrelevant.

Powered by Editorial Manager® and ProduXion Manager® from Aries Systems Corporation

*** Response: We thank the reviewer for pointing that out. We deleted this part of the sentence.

Line 198: Change “studies” to “study”

*** Response: We changed “studies” to “study”.

Line 220: Everywhere else in the manuscript you say “24” studies.

*** Response: This was a typographical error. It has been corrected to 24.

Lines 287-290: This belongs in the next section, not here.

*** Response: We thank the reviewer for its feedback on the matter. However, without further justifications as to why this belong in the next section (next paragraph or in the results?), we feel confident that it does belong where it is now and would prefer that it remains that way.

Lines 357-359: You call a “P = 0.07” “marginally significant” below.

*** Response: We rectified our statement regarding the p-value of 0.07 stating it was not statistically significant, but that visual assessment could detect a potential trend where studies that were considered as gradients were most often associated with negative effect sizes (not supported statistically; lines 408-413).

Lines 362-365: How is the “true heterogeneity” compared to the “methodological variability” assessed? Is this left-over variance not explained by your model?

*** Response: The methodological variability is the heterogeneity due to sampling error within each individual study included in the meta-analysis, whereas the true heterogeneity is due to specific study characteristics, which can introduce heterogeneity among the effect sizes. The combination of this methodological and true heterogeneity is referred to as total heterogeneity. We tried to clear that up in the methodology section (lines 279-290) so that lines 362-365 are easier to understand.

Line 366: Change “will be” to “was”.

*** Response: We changed “will be” to “was”.

Lines 369: You mean hydropower, dams, and reservoirs DID have a statistically significant effect on macroinvertebrate richness, right?

*** Response: Yes, this has been rectified.

Line 399: Change “little” to “few”.

*** Response: We changed “will be” to “was”.

Line 404: add “for all levels” after “df”

*** Response: We added “(for all levels)” after “df”.

Lines 448-450: Sentence begins in a way redundant with previous sentence. Change “Hydropower impacts macroinvertebrates, their richness…” to “Macroinvertebrate richness…”

*** Response: This has been rectified.

Line 452: Change “from which” to “with”

*** Response: We modified “from which” to “with” as well as “showed” to “showing” in the remainder of the sentence.

Powered by Editorial Manager® and ProduXion Manager® from Aries Systems Corporation

	Lines 467: Why are you now calling this a marginal effect?<br><br>*** Response: This is a leftover typographical error from a previous version of this manuscript, we thank the reviewer for pointing it out. We deleted part of the sentence mentioning the “marginality” of the mean effect size which is not valid for the updated/enhanced meta-analysis (17 to 24 studies included).<br><br>Lines 472: Delete “and thus potentially strengthen the statistical significance of the result”. Awkward phrasing that doesn’t seem necessary.<br><br>*** Response: We thank the reviewer for its output, we deleted this part of the sentence.<br><br>Line 516: Change “been causing” to “caused”.<br><br>*** Response: We changed “been causing” to “caused”.<br><br>Lines 539: Delete “(none could be included in this case)”. Redundant.<br><br>*** Response: We deleted this parenthesis.<br><br>6. PLOS authors have the option to publish the peer review history of their article (what does this mean?). If published, this will include your full peer review and any attached files.<br><br>If you choose “no”, your identity will remain anonymous but your review may still be made public.<br><br>Do you want your identity to be public for this peer review? For information about this choice, including consent withdrawal, please see our Privacy Policy.<br><br>Reviewer #1: No<br><br>Reviewer #2: No
**Additional Information:**	
**Question**	**Response**
**Financial Disclosure**<br><br>Enter a financial disclosure statement that describes the sources of funding for the work included in this submission. Review the submission guidelines for detailed requirements. View published research articles from *PLOS ONE* for specific examples.<br><br>This statement is required for submission and **will appear in the published article** if the submission is accepted. Please make sure it is accurate.	MM and GT were funded by the Natural Sciences and Engineering Research Council of Canada (NSERCC). GT was also funded by the Fonds Qubcois de la Recherche sur la Nature et les Technologies (FQRNT), Fondation Polytechnique and Hydro-Quebec, as well as the Institut de l’Environnement, le Developpement Durable et l'economie Circulaire (EDDEC) and Banque TD. The funders had no role in study design, data collection and analysis, decision to publish, or preparation of the manuscripts.

**Unfunded studies**<br>Enter: *The author(s) received no specific funding for this work.*<br><br>**Funded studies**<br>Enter a statement with the following details:<br>• Initials of the authors who received each award<br>• Grant numbers awarded to each author<br>• The full name of each funder<br>• URL of each funder website<br>• Did the sponsors or funders play any role in the study design, data collection and analysis, decision to publish, or preparation of the manuscript?<br>• **NO** - Include this sentence at the end of your statement: *The funders had no role in study design, data collection and analysis, decision to publish, or preparation of the manuscript.*<br>• **YES** - Specify the role(s) played.<br><br>* typeset	
**Competing Interests**<br><br>Use the instructions below to enter a competing interest statement for this submission. On behalf of all authors, disclose any competing interests that could be perceived to bias this work—acknowledging all financial support and any other relevant financial or non-financial competing interests.<br><br>This statement is required for submission and **will appear in the published article** if the submission is accepted. Please make sure it is accurate and that any funding sources listed in your Funding Information later in the submission form are also declared in your Financial Disclosure statement.<br><br>View published research articles from *PLOS ONE* for specific examples.	The authors have declared that no competing interests exist.

**NO authors have competing interests**<br><br>Enter: *The authors have declared that no competing interests exist.*<br><br>**Authors with competing interests**<br><br>Enter competing interest details beginning with this statement:<br><br>*I have read the journal's policy and the authors of this manuscript have the following competing interests: [insert competing interests here]*<br><br>* typeset	
**Ethics Statement**<br><br>Enter an ethics statement for this submission. This statement is required if the study involved:<br><br>• Human participants<br>• Human specimens or tissue<br>• Vertebrate animals or cephalopods<br>• Vertebrate embryos or tissues<br>• Field research<br><br>Write "N/A" if the submission does not require an ethics statement.<br><br>General guidance is provided below. Consult the submission guidelines for detailed instructions. **Make sure that all information entered here is included in the Methods section of the manuscript.**	N/A

**Format for specific study types**<br><br>**Human Subject Research (involving human participants and/or tissue)**<br>• Give the name of the institutional review board or ethics committee that approved the study<br>• Include the approval number and/or a statement indicating approval of this research<br>• Indicate the form of consent obtained (written/oral) or the reason that consent was not obtained (e.g. the data were analyzed anonymously)<br><br>**Animal Research (involving vertebrate animals, embryos or tissues)**<br>• Provide the name of the Institutional Animal Care and Use Committee (IACUC) or other relevant ethics board that reviewed the study protocol, and indicate whether they approved this research or granted a formal waiver of ethical approval<br>• Include an approval number if one was obtained<br>• If the study involved *non-human primates*, add *additional details* about animal welfare and steps taken to ameliorate suffering<br>• If anesthesia, euthanasia, or any kind of animal sacrifice is part of the study, include briefly which substances and/or methods were applied<br><br>**Field Research**<br><br>Include the following details if this study involves the collection of plant, animal, or other materials from a natural setting:<br>• Field permit number<br>• Name of the institution or relevant body that granted permission	
**Data Availability**<br><br>Authors are required to make all data underlying the findings described fully available, without restriction, and from the time of publication. PLOS allows rare exceptions to address legal and ethical concerns. See the PLOS Data Policy and FAQ for detailed information.	Yes - all data are fully available without restriction

Powered by Editorial Manager® and ProduXion Manager® from Aries Systems Corporation

A Data Availability Statement describing where the data can be found is required at submission. Your answers to this question constitute the Data Availability Statement and **will be published in the article**, if accepted.<br><br>**Important:** Stating 'data available on request from the author' is not sufficient. If your data are only available upon request, select 'No' for the first question and explain your exceptional situation in the text box.<br><br>Do the authors confirm that all data underlying the findings described in their manuscript are fully available without restriction?	
**Describe where the data may be found in full sentences. If you are copying our sample text, replace any instances of XXX with the appropriate details.**<br><br>• If the data are **held or will be held in a public repository**, include URLs, accession numbers or DOIs. If this information will only be available after acceptance, indicate this by ticking the box below. For example: *All XXX files are available from the XXX database (accession number(s) XXX, XXX.)*.<br>• If the data are all contained **within the manuscript and/or Supporting Information files**, enter the following: *All relevant data are within the manuscript and its Supporting Information files.*<br>• If neither of these applies but you are able to provide **details of access elsewhere**, with or without limitations, please do so. For example:<br><br>*Data cannot be shared publicly because of [XXX]. Data are available from the XXX Institutional Data Access / Ethics Committee (contact via XXX) for researchers who meet the criteria for access to confidential data.*<br><br>*The data underlying the results presented in the study are available from (include the name of the third party*	All relevant data are within the manuscript and its Supporting Information files.

and contact information or URL).<br>• This text is appropriate if the data are owned by a third party and authors do not have permission to share the data.<br><br>* typeset	
Additional data availability information:	

CIRAIG – POLYTECHNIQUE MONTRÉAL

Gabrielle Trottier, PhD
gabrielle.trottier@polymtl.ca
CIRAIG – Polytechnique Montréal
3333 Queen-Mary Rd, suite 310
Montréal, Québec, H3V 1A2
Tel: 1-514-677-9137

Dear M. Jay Richard Stauffer, Jr.

July 28th, 2022

(Academic Editor – PLOS ONE),

As per your invitation, we are re-submitting a revised version of the manuscript "The impacts of hydropower on freshwater macroinvertebrate richness: A global meta-analysis" by G. Trottier, K. Turgeon, D. Boisclair, C. Bulle and M. Margni for consideration as a meta-analysis research paper in your journal. We thank the two reviewers and the academic editor in charge of this manuscript for their meaningful comments and suggestions to improve this manuscript. Considering these comments and suggestions, we made numerous modifications and corrections to the manuscript.

We provided further justification for the use of species richness and grabs/nets as sampling gear in this study. We corrected the discrepancy between the number of papers used in this meta-analysis (24 studies is the right number, 17 studies were in the earlier versions of this manuscript). We clarified the role of moderators regarding heterogeneity in effect sizes and developed the reasoning behind the potential effect of a latitudinal gradient on species richness effect sizes. As per reviewer 2's recommendation, we deleted a paragraph describing the meta-analytic framework, which felt like too many details for PLOS ONE targeted audience. We also improved clarity in the section regarding heterogeneity analysis in meta-analyses. Finally, we subjected the manuscript to numerous minor edits and corrected grammatical mistakes, as per reviewers' recommendations.

Please find below a rebuttal letter that responds to each point raised by the academic editor and the two reviewers and, in attachment, the revised manuscript with track changes, as well as an unmarked version of the revised manuscript.

We thank you in advance for considering this version of the manuscript for publication in your journal.

Sincerely,

Gabrielle Trottier, PhD

Manuscript

Click here to access/download;Manuscript;Manuscript_Trottier.etal_UPDATE

The impacts of hydropower on freshwater macroinvertebrate richness: A global meta-analysis

Gabrielle Trottier[1*], Katrine Turgeon[2], Daniel Boisclair[3], Cécile Bulle[4] and Manuele Margni[1,5]

[1] CIRAIG, Département de mathématique et génie industriel, Polytechnique Montréal, Montréal, Québec, Canada
*Corresponding author: gabrielle.trottier@polymtl.ca, 514-340-4711 #2136

[2] ISFORT, Université du Québec en Outaouais, Ripon, Québec, Canada
katrine.turgeon@uqo.ca

[3] Département de sciences biologiques, Université de Montréal, Montréal, Québec, Canada
daniel.boisclair@umontreal.ca

[4] CIRAIG, Département de stratégie, responsabilité sociale et environnementale, École des sciences de la gestion, Université du Québec à Montréal, Montréal, Québec, Canada
bulle.cecile@uqam.ca

[5] Institute of Sustainable Energy, HES-SO Valais-Wallis, Sion, Switzerland
manuele.margni@polymtl.ca

Abstract

Hydroelectric dams and their reservoirs have been suggested to affect freshwater biodiversity. However, studies investigating the consequences of hydroelectric dams and reservoirs on macroinvertebrate richness have reached opposite conclusions. We carried out a meta-analysis devised to elucidate the effects of hydropower, dams and reservoirs on macroinvertebrate richness while accounting for the potential role played by moderators such as biomes, impact types, study designs, sampling seasons and gears. We used a random/mixed-effects model, combined with robust variance estimation, to conduct the meta-analysis on 107 pairs of observations (*i.e.*, impacted versus reference) extracted from 24 studies (more than one observation per study). Hydropower, dams and reservoirs did significantly impact macroinvertebrate richness in a clear, directional and statistically significant way, where macroinvertebrate richness in hydropower, dams and reservoirs impacted environments were significantly lower than in unimpacted environments. ~~However~~, we also observed a large range of effect sizes, from very negative to very positive impacts of hydropower. We tried to account for the large variability in effect sizes using moderators, but none of the moderators included in the meta-analysis had statistically significant effects. This suggests that some other moderators (unavailable for the 24 studies) might be important (*e.g.*, temperature, granulometry, wave disturbance and macrophytes) and that macroinvertebrate richness may be driven by local, smaller scale processes. As new studies become available, it would be interesting to keep enriching this meta-analysis, as well as collecting local habitat variables, to see if we could statistically strengthen and deepen the conclusions of this meta-analysis.

Introduction

Freshwater ecosystems are vital resources for humans and support a biota that is rich, sensitive and characterized by a high level of endemicity [1]. Ecosystems functions and integrity often depend on biodiversity, which can be described by three indices: species richness (*i.e.*, number of species), community assemblage (*i.e.*, proportions of different species or taxonomic groups in the community) and functional diversity (*i.e.*, variability in organisms' traits that can influence ecosystem functioning; [1]). For millennia, humans have used freshwater ecosystems, through water extraction for drinking and irrigation purposes, water regulation for hydropower production, flood control and recreation [1], but these usages often come with a cost on freshwater ecosystems biodiversity [2,3].

Hydroelectric dams and the creation of reservoirs, at all stages of their life cycle (*i.e.*, from the construction, to operation and decommission of a dam), can affect freshwater biodiversity [4]. Dams create a physical barrier, which can impair the natural flow of water, sediments and nutrients [5,6] and limit the movement of organisms [7]. The alteration of the natural hydrological regime can affect freshwater biodiversity through various biological mechanisms (*i.e.*, mortality through desiccation, mismatch timing in life history strategies, lotic to lentic community changes, reduction/extirpation of endemic and specialist species; [8,9]) and through degraded water quality (anoxic or hypoxic releases [dissolved oxygen], hypolimnetic or epilimnetic releases [temperature], pH, organic carbon, turbidity; [4,10–13]).

Studies investigating the impact of hydropower on macroinvertebrate richness drew contrasting conclusions. Some studies reported that macroinvertebrate richness is negatively impacted by hydropower, through general flow regulation [14–16] and water

level fluctuation (or drawdown; [17–22]). Others observed higher macroinvertebrate richness downstream of a dam [23,24] or in regulated rivers (as opposed to natural ones; [25]). Finally, Marchetti et al. [26] found little difference between impacted flows (*i.e.*, dam-induced permanent low flow) and "natural-like" flows (*i.e.*, high flows in winter and spring, low flows in summer and falls). A meta-analysis could elucidate patterns and interactions that may exist between hydropower, macroinvertebrate richness, and the context in which studies have been conducted.

Many challenges can be encountered when conducting a meta-analysis, and many ecological facets of studied ecosystems can influence the magnitude and significance of human impacts [27], along with study-specific methodological characteristics (*e.g.*, different study design). The influence and the variability brought about by these facets and characteristics can be accounted for through variables, also called moderators in meta-analysis [28]. For instance, the location of each study site can influence the observed effects [27,29,30]. A latitudinal biodiversity gradient is a good example of the influence of spatial location. Species richness is known to be highest in the tropics and lowest at the poles [30,31]. Thus, losing few species to hydropower activities will not have the same repercussions on low diversity macroinvertebrate communities than higher diversity communities (*e.g.*, losing 2/10 species [20% loss] versus 2/25 species [8% loss]). Hydropower can lead to different types of impacts. A study can analyze the impacts of hydropower upstream of a dam, that is in the reservoir, or downstream of a dam. Impacts also varies depending on the type of water management in place, storage reservoir with winter water level drawdown, hydropeaking or typical run-of-river hydrological regime. These variations in studies, along with the location under study, can introduce variability

and heterogeneity in the results, which can be accounted for through moderators. The experimental design can also influence how human-induced impacts magnitude are reflected in a study [27]. As demonstrated in Christie et al. [32], different sampling designs may affect the conclusion of a study. Using simulations, they demonstrated that Before-After (BA), Control-Impact (CI; analogous to space-for-time substitution) and After designs are far less accurate than Randomized Controlled Trials (RCT) and Before-After Control-Impact (BACI) designs. RCT and BACI are much harder to implement in ecology because true randomization can be difficult with larger scale designs and getting data before the impacts or human intervention is sometimes impossible. Thus, we must account for the effect of the experimental design on a study outcome, especially in a meta-analysis, where the effects of multiple different studies are combined. Sampling season can also influence the results across studies, as macroinvertebrate communities differs in terms of abundance and diversity throughout the year (*i.e.*, maximum diversity in late summer and autumn versus underrepresented diversity in spring and early/mid-summer; [33]). At a more local scale, the habitat stratum that is sampled is also most likely to influence the results [27], especially when studying macroinvertebrates. These organisms possess characteristics that make them highly adapted to their environment [34] and because lakes, reservoirs and river beds are so heterogeneous, macroinvertebrates are often patchily distributed, requiring extensive sampling [35]. Thus, the type of sampling gear used to sample will likely affect the type of organisms inventoried in each study. However, using sampling gear as a proxy for habitat stratum might need a caveat as many biomonitoring protocols (*e.g.*, United State Environmental Protection Agency) use nets for multiple-

habitat assessments. In this study, we assumed that different sampling methods would collect different macroinvertebrate communities.

The objective of this manuscript is to conduct a meta-analysis about the impacts of hydropower dams and their reservoirs on macroinvertebrates richness while accounting for a series of moderators defining the context of the studies included. The moderators included in this manuscript are the following: 1) biomes (*i.e.*, boreal, temperate, and tropical), a proxy for location/latitudinal gradient, 2) type of impact, which is reflected by the position of a sample in relation to the dam (*i.e.*, upstream or downstream of the dam). Downstream stations are impacted by a reduced flow and hydropeaking dynamics, whereas upstream stations are impacted by drawdown and water level fluctuations due to reservoir management, 3) type of study designs such as cross-sectional (*i.e.*, reference natural lake versus impacted reservoir) and longitudinal spatial gradient (*i.e.*, upstream of a dam [reservoir] versus downstream of a dam [river]), which are two different variants of CI study design, 4) sampling seasons (*i.e.*, spring, summer, fall, winter; we were interested in the coarser temporal effect of seasons rather than daily changes in colonization following water regulation) and 5) sampling gears, a proxy for habitat stratum (*i.e.*, grabs and nets). Even if richness constitutes only one of three components of biodiversity, we chose to focus on macroinvertebrate richness because it is easier to quantify and extract from the scientific literature than community composition and also a lot more common than functional diversity.

Methodology

Achieving our objective requires to first establish a research strategy, second to do a data collection comprising information regarding macroinvertebrate richness in

hydropower impacted habitat versus reference ones, biome, the type of impact, study design, sampling seasons and gear. Third, it requires to compute effect sizes for each study and finally, combine them to assess if the mean effect size is significantly different from zero [36] and if the presence of other moderators can influence these results.

Research strategy

In this study, we used the PRISMA (Preferred Reporting Items for Systematic and Meta-Analyses) methodology, flow diagram (Fig 1) and checklist (S1 Table) proposed by Moher et al. [37], to report systematic literature reviews and meta-analyses. A systematic literature review was conducted using the Web of Science Core Collection database, which includes all journals indexed in Science Citation Index Expanded (SCI-EXPANDED), Social Sciences Citation Index (SSCI), Arts & Humanities Citation Index (A&HCI), Conference Proceedings Citation Index – Science (CPCI-S), Conference Proceedings Citation Index – Social Science & Humanities (CPCI-SSH) and Emerging Sources Citation Index (ESCI; [38]). The research strategy was constrained between 1989 (earliest searchable year) and 2021 and contained a combination of the four following field of research: 1) hydropower (hydropower OR hydroelectric* OR dam OR dams OR reservoir* OR impound* OR run-of-river OR "run of river" OR drawdown* OR hydropeak* OR dam* OR "water level fluctuation" OR "water-level fluctuation" OR "water level variation" OR "water-level variation" OR "water level regulation" OR "water-level regulation" OR "water level manipulation" OR "water-level manipulation" OR "water management"), 2) biodiversity (biodiversity OR richness), 3) freshwater ecosystems (freshwater OR aquatic) and 4) aquatic insects (*invertebrate* OR *benth* OR insect* OR arthropod*). Research strategy also excluded all studies pertaining to beaver and

agricultural dams (NOT beaver* NOT agricult*). The research strategy resulted in 518 research articles, as per September 1st 2021.

INSERT FIG 1 HERE

Fig 1. PRISMA diagram. Specific to this meta-analysis, from Moher et al. [37].

The results were extracted as a list to further evaluate the relevance of every study based on a list of criteria. The following criteria were applied to assess the inclusion of any study in the meta-analysis: 1) the study had to refer specifically to hydropower related impacts (*i.e.*, reservoir, run-of-the-river or hydropeaking, multi-purpose reservoirs were also checked for hydropower impacts), 2) the scope of the study had to address freshwater ecosystems and macroinvertebrates and 3) the study had to be empirical (*i.e.*, excluding literature reviews, modelling exercises) and provide an explicit richness, error and sample size value for both a reference and impacted site (*i.e.*, cross-sectional studies [reference versus impacted]) or gradient of impact (longitudinal spatial gradient studies [upstream of the dam/reservoir versus one or multiple sites downstream of the dam]). Out of the 518 research studies that resulted from the research strategy, only 24 met the above criteria (see geographical disposition of studies in Fig 2).

INSERT FIG 2 HERE

Fig 2. World map showing the geographical disposition of the studies used in this meta-analysis. (1) Aroviita and Hämäläinen (2008) *[39]*, (2) Valdovinos et al. (2007) *[20]*, (3) Marchetti et al. (2011) *[26]*, (4) Molozzi et al. (2013) *[40]*, (5) Takao et al. (2008) *[15]*, (6) Kullasoot et al. (2017) *[16]*, (7) White et al. (2011) *[22]*, (8) Smokorowski et al. (2011) *[25]*, (9) Englund and Malmqvist (1996) *[18]*, (10) Jackson et al. (2007) *[14]*, (11) Kraft (1988) *[17]*, (12) Mellado-Dìaz et al. (2019) *[41]*, (13) Bruno et al. (2019) *[42]*, (14) Milner et al. (2019) *[43]*, (15) Steel et al. (2018) *[44]*, (16) Schneider and Petrin (2017) *[45]*, (17) Vaikasas et al. (2013) *[46]*, (18) Doledec et al. (2021) *[47]*, (19) Wang et al. (2016) *[48]*, (20) Nukazawa et al. (2020) *[49]*, (21) Quadroni et al. (2020) *[50]*, (22) Vilenica et al. (2020) *[51]*, (23) Uieda and Marcal (2020) *[52]* and (24) Cazaubon and Giudicelli (1999) *[53]*.

Data collection

Richness metrics and moderators were collected for each of these 24 studies by the corresponding author (S2 and S3 Tables). We extracted richness (*i.e.*, number of taxa),

error measure (*e.g.*, standard deviation), sample size (*i.e.*, number of reference observations and impacted observations used to compute richness and its associated error) and a suite of moderators such as biome (*i.e.*, boreal, temperate and tropical; [54]), type of impact (*i.e.*, water level fluctuations upstream, due to reservoir management, or flow regulation downstream due to dam operations), type of study, (*i.e.*, cross-sectional [reference natural lake versus impacted reservoir] or longitudinal spatial gradient [reference; upstream of the dam/reservoir versus impacted; downstream of the dam]), sampling season (*i.e.*, spring [March to May], summer [June to August], fall [September to November] and winter [December to February], according to the hemisphere) and sampling gear (*i.e.*, net, grab or colonization basket). These moderators were chosen based on the availability of said moderators in each of the 24 studies, their potential influence on macroinvertebrate richness and complemented with expert judgement. In studies where meaningful data were presented exclusively in graphical format, values were extracted using Engauge Digitizer 10.4 [55].

Effect size

To compute effect sizes, we calculated the standardized mean differences, also called Cohen's *d* – which expresses the distance between two means (*i.e.*, impact and reference) in terms of their common standard deviation [56]. For most studies – except Takao et al. [15], Schneider and Petrin [45] and Vilenica et al. [51], we computed at least two effect sizes per study, leading to a total of 107 effect sizes, with a certain level of within-study dependency (*i.e.*, effect sizes in one study are not entirely independent from each other). Cohen's *d* is calculated as per equation 1 [56]:

$$d = \frac{\bar{X}_1 - \bar{X}_2}{\sqrt{\left(\frac{(n_1 - 1)s_1^2 + (n_2 - 1)s_2^2}{n_1 + n_2 - 2}\right)}} \tag{1}$$

where $\bar{X}_1$ and $\bar{X}_2$ are the mean richness, s_1^2 and s_2^2 are the standard deviation (SD) and, n_1 and n_2 are the number of observations used to compute the mean and SD for impacted and reference samples, respectively. The common variance (V_d) associated with the effect size (*d)* is calculated using equation 2:

$$V_d = \frac{n_1 + n_2}{n_1 \cdot n_1} + \frac{d^2}{2(n_1 + n_2)} \tag{2}$$

In the case of smaller sample size (usually < 20 studies), a correction factor is applied to Cohen's *d* to reduce the positive bias (negligible with bigger sample size) and to provide a better estimate. The corrected effect size is then called a Hedges' g [57]. A small sample correction factor (J) was computed using equation 3:

$$J = 1 - \frac{3}{4df - 1} \tag{3}$$

where df refers to the degrees of freedom ($n_1 + n_2 - 2$). Thus, the corrected effect size *g* and variance V_g are calculated following equations 4 and 5, respectively:

$$g = J \cdot d \tag{4}$$

$$V_g = J^2 \cdot V_d \tag{5}$$

A positive g means the impacted environment or sample has more richness in comparison to a reference environment or sample. The *metafor* package [28] was used to compute the effect sizes and sampling variances (*i.e.*, *escalc* function) and the *ggplot2* package [58] was used to graphically visualize the results of the meta-analysis.

Data analysis

Publication bias

Studies with large significant results are more likely to be published than studies with non-significant results, this is called publication bias [36]. A funnel plot was used to evaluate the presence of a publication bias in the meta-analysis [59], and a regression test for funnel plot was used to detect potential asymmetry (*i.e.*, if only large significant studies, range of outcomes is not well represented and studies with non-significant results might not even be present; [60]). The *metafor* package [28] was used for asymmetry analysis (*i.e.*, *funnel* and *regtest* functions).

Heterogeneity

There are two sources of heterogeneity in a meta-analysis 1) the heterogeneity due to sampling error, or the within-study heterogeneity (*i.e.*, methodological heterogeneity), which is always present in meta-analyses as every study uses different samples, and 2) the true heterogeneity due to specific study characteristics (*e.g.*, biome) and dissimilarities in methodologies among studies (*e.g.*, study design), which can introduce variability among true effect sizes [28,61].The combination of the methodological and the true heterogeneity is referred to as the total heterogeneity. It is interesting to examine this total heterogeneity and identify the different moderators and their relative contributions to the magnitude and direction of these effect sizes [36]. We evaluated the statistical significance and the magnitude of the total heterogeneity of effect sizes among studies (*i.e.*, heterogeneity analysis) using the Q statistic, followed by the I^2 index to identify how much of this total heterogeneity is due to true heterogeneity [61]. The *metafor* package [28] was used for heterogeneity analysis (*i.e.*, *rma* function).

Random/mixed-effects model: dealing with heterogeneity

Two types of meta-analytic models can be used in a meta-analysis, fixed-effects or random/mixed-effects. A fixed-effects model considers only the studies included in the meta-analysis and within study sampling variability, not between studies [36]. No inference can be made outside this set of studies (*i.e.*, conditional inferences; [28]). A random/mixed-effects model considers the set of analyzed studies as a sample of a larger population of studies [28]. Thus, it allows the researcher to make inferences regarding what would be found if an entire new meta-analysis, with a different set of studies, was performed (*i.e.*, unconditional inferences; [28]). It also allows two sources of variation, within and among studies [36]. Such an approach is especially appropriate when dealing with heterogeneity among studies [61] and, with a random/mixed-effects model approach, it is also possible to include moderators, which can account for some of that heterogeneity [28]. It is important to highlight that these moderators do not impact species richness, they rather explain additional heterogeneity in the meta-analysis effect sizes. Here, a random/mixed-effects modelling is preferred to a fixed-effects modelling approach since 1) a significant amount of heterogeneity was found in the previous heterogeneity analysis and 2) because it offers the possibility to model and explain some of that heterogeneity using moderators [56].

Robust variance estimation: dealing with dependency

If our effect sizes were all independent from each other, we could have simply used a random/mixed-effects model. However, because this meta-analysis is dealing with multiple effect sizes per study, where observations are not methodologically and spatially independent from each other, it is inappropriate to use a regular meta-analysis approach (*i.e.*, random/mixed-effects model), where the effect sizes are assumed to be independent.

One way to account for dependency of effect sizes is to combine the random/mixed-effects model with the robust variance estimation (RVE) method. The RVE estimates the overall effect size over studies using a weighted mean of the observed effect sizes [62]. It doesn't require knowledge about the within-study covariance, it can be applied to any type of dependency and effect sizes, it simultaneously accommodates for multiple sources of dependencies, it does not require the effect sizes to comply to any particular distribution assumptions, it leads to unbiased fixed-effects and standard errors estimates and can also give an estimate of among-study variance [62,63]. Because the most common source of dependence within the effect sizes in this meta-analysis is the correlated nature of the observations (*i.e.*, multiple measures within a study; methodological and spatial correlation) and not the hierarchical nature (*i.e.*, common nesting structure between studies; a sample within a transect, within site and within a lake), a correlated effects weighting method was used [63]. Thus, we used a RVE based on a correlated effects model and adjusted for small sample size (< 40 studies; [63]). Finally, a sensitivity analysis was computed to assess the effect of a varying rho (ρ) value, which is a user-specified value of the within-study effect sizes correlation (*i.e.*, the correlation between two samples taken in the same water body in one specific study – spatial and methodological correlation; [63]). The *robumeta* package [63] was used to fit the RVE meta-regression model (*i.e.*, *robu* function) and to compute the sensitivity analysis (*i.e.*, *sensitivity* function). All statistical analyses were made using R version 3.0.2 [64].

Results

Methodological results

The purpose of this first set of results is to validate our methodological approach and choices, they will not be the subject of discussion. No statistical asymmetry was observed in the funnel plot (z = -1.74; P = 0.08; S1 Fig), a wide range of results and significance levels were represented by the studies included in this meta-analysis. The total heterogeneity among the effect sizes was statistically significant ($Q_{df=23}$ = 140.16; P < 0.0001), which indicated greater total heterogeneity than expected by the sampling error alone. The estimated amount of this total heterogeneity among the effect sizes was T^2 = 2.01; 95% confidence interval [CI] = 1.05-4.90. Of that total heterogeneity, a large amount (I^2 = 89.44%; CI = 81.59-95.39) was due to true heterogeneity between the studies, rather than just methodological heterogeneity. Thus, further examination of the true heterogeneity is warranted and was done through the analysis of multiple moderators.

Meta-analysis results

The meta-analysis of 24 studies (107 pairs of observations; S1 Table) suggests that hydropower dams and reservoirs did have a statistically significant effect on macroinvertebrate richness. The mean effect size (*i.e.*, Hedge's *g*) estimate of our RVE model was -0.84 (95% CI = -1.62 to -0.05; P = 0.04), without accounting for the different moderators (Fig 3). The large confidence interval not overlapping zero indicates that the mean effect size is statistically significant, but also highlights a wide range of effect sizes across studies. The sensitivity analysis shows that the estimates of the mean effect size and standard errors, as well as the estimate of between-study variance in study-average effect sizes (τ^2), are relatively insensitive to different value of ρ (S4 Table).

INSERT FIG 3 HERE

Fig 3. Forest plot of the meta-analysis. The mean effect size is -0.84 (95% CI = -1.62 to -0.05, shaded grey area), where study type is shape-coded (*i.e.*, circle for longitudinal studies and squares for cross-sectional studies) and biome color

coded (*i.e.*, boreal in blue, temperate in yellow and tropical in red). A negative effect size means that there is a negative impact of hydropower in impacted sites as opposed to reference sites, whereas a positive effect size means that there is positive impact of hydropower in impacted sites as opposed to reference sites.

Moderators had very little influence in the effects of dams and reservoirs on macroinvertebrate richness. Biome did not significantly explain variability effects sizes. For this moderator, it was only possible to make statistical interpretation for the temperate level (estimate = -0.23; 95% CI = -1.97 to 1.51; P = 0.74; Fig 4A) and tropical level (estimate = 0.02; 95% CI = -4.53 to 4.57; P = 0.99; Fig 4A). We can interpret with statistical confidence that temperate and tropical biome did not significantly differ from zero. The boreal level had too few degrees of freedom ($df_s < 4$), which invalidates the Satterthwaithe approximation (*i.e.*, calculation of the effective df_s of a linear combination of independent sample variances; [63,65,66]). Whether it is significant or not, we cannot interpret the boreal result with strong statistical confidence. Thus, the results of the RVE with this moderator in the equation have to be interpreted with caution. On the contrary, both type of impact and study moderators had enough df_s (for all levels) for robust statistical analysis. Statistically significant effects were neither found for downstream flow regulation (estimate = -0.50; 95% CI = -1.24 to 0.25; P = 0.18) nor for upstream water level fluctuations/drawdown impact types (estimate = -1.20; 95% CI = -3.45 to 1.05; P = 0.26; Fig 4B). When study type was used as a moderator, there was no significant difference in effect sizes for cross-sectional design studies (*i.e.*, natural versus impacted; estimate = 0.45; 95% CI = -1.14 to 2.03; P = 0.56) and longitudinal gradient type studies (*i.e.*, spatial gradient; estimate = -1.11; 95% CI = -2.32 to 0.10; P = 0.07; Fig 4C). However, we can observe a visual trend where studies that were considered as gradients were most often associated with negative effect sizes (not supported statistically). As for results from the season moderator, conclusions can only be drawn for the summer and winter levels.

Statistically significant effects were neither found for summer (estimate = -0.46; 95% CI = -2.26 to 1.24; P = 0.58) nor for winter (estimate = -0.39; 95% CI = -2.83 to 2.05; P = 0.70; Fig 4D). Results are similar for the sampling gear moderator, statistical inference can only be drawn for the grab level, where no statistically significant effect can be observed (estimate = -0.67; 95% CI = -8.18 to 6.85; P = 0.83; Fig 4E), other levels cannot be interpreted with confidence due to insufficient df_s (Satterthwaithe approximation invalidated).

INSERT FIG 4 HERE

Fig 4. Plots showing the mean effect sizes and their confidence interval for each of the moderators. Value in black is the mean effect size of the meta-analysis and the other colors are related to the different effect sizes when including specific moderators. When in grey, statistical significance of moderator cannot be interpreted with confidence due to statistical power issues (df_s insufficient). When effect size is in color (*i.e.*, blue) statistical interpretation can be made with confidence, whether it is significant or not (sufficient df_s). Asterisk signifies statistically marginally significant effect.

Discussion

The meta-analysis conducted on 107 pairs of observations (*i.e.*, impacted versus reference) extracted from 24 studies suggests that hydropower does statistically impact macroinvertebrate richness in a clear directional way. Macroinvertebrates richness in hydropower, dams and reservoirs impacted environments is significantly lower than in unimpacted environments (Fig. 2). To our knowledge, this meta-analysis is the first to resolve the result divergence observed in the existing literature. In fact, more than 88% of the effect sizes were significantly different from zero with 56% of the observations showing reduced richness due to hydropower and 32% of the observations showing reduced richness in reference conditions. These percentages, along with the statistically significant mean effect size (*i.e.*, -0.84; 95% CI = -1.62 to -0.05; P = 0.04), suggest a

predominance of reduced macroinvertebrate richness due to hydropower, dams and reservoirs.

~~However,~~ we also observed a large heterogeneity in macroinvertebrate richness responses to hydropower (Fig 3). Part of this variability across observations and studies can usually be explained by environmental and methodological variability, which we can try to control using moderators. In our meta-analysis, we considered biome (*i.e.*, boreal, temperate or tropical), type of impact (*i.e.*, water level fluctuations in the reservoir or flow regulation downstream of the dam), the type of study (*i.e.*, spatial longitudinal gradient [upstream vs downstream] or cross-sectional [natural lake versus impacted reservoir), sampling season (*i.e.*, spring, summer, fall and winter) and sampling gear (*i.e.*, net, grab and colonization basket) as moderators. Despite the documented effects on macroinvertebrate richness of the moderators included in our analysis [27,29,30,32,33], none of our moderators statistically explained heterogeneity in macroinvertebrate richness responses to hydropower.

Biome moderator

In general, it is quite common to observe a latitudinal gradient in species richness, where there is a maximum richness in the tropics and a decline towards the poles [30,31,67,68]. In a meta-analysis, Turgeon et al. [69], showed that the impacts of impoundments on fish richness was different across biomes. Significant declines in richness were observed in the tropics, a lower decline was observed in temperate regions and no impacts was observed in boreal biomes. Thus, we hypothesized that a latitudinal gradient could also influence the mean effect size in macroinvertebrates (*e.g.*, losing one species over a few species is more costly in terms of biodiversity loss, than over multiple

species). Our data did not support a latitudinal trend which is not too surprising because there is no clear pattern about whether or not macroinvertebrate richness follows a latitudinal gradient [70–72]. Pearson and Boyero [73] observed a richness peak around the equator for dragonflies (*i.e.*, odonata), but no clear global pattern for caddisflies (*i.e.*, trichoptera), whereas Vinson and Hawkins [71] observed richness peaks at mid-latitudes in South and North America for mayflies (*i.e.*, ephemeroptera), stoneflies (*i.e.*, plecoptera) and caddisflies (*i.e.*, trichoptera; [EPT] orders). On the contrary, Scott et al. [74] did not observe this latitudinal gradient for EPT in northern Canada. Geographic range (*i.e.*, narrow range and exclusion of extreme latitudes; [71]), sampling effort [75], macroinvertebrate specific life-history strategies [73,74] and data resolution (*i.e.*, weak gradient for local species richness; [76]) were probably the reason behind this lack of consensus [77], and absence of a trend in this meta-analysis. Moreover, the unbalanced sample size across biomes may have impeded any clear conclusions. Boreal samples represented 9% of the data, tropical samples 11% and roughly 79% of the data belong to temperate ecosystems. This prevented us from statistically supporting a trend for both boreal and tropical observations.

Impact type moderator

Water level fluctuation in reservoirs is characterized by yearly drawdown (*i.e.*, long-term oscillations in the water level), whereas flow regulation usually is reflected by daily or weekly changes in flow downstream of a dam (*i.e.*, short-term oscillations; [20]), keeping in mind that variation in the reservoir are also reflected downstream. In reservoir experiencing water level fluctuations in the form of winter drawdown (21% of the reservoirs in this meta-analysis; littoral zone samples), the littoral zone is exposed to

desiccation and freezing for an extended period of time, which caused a loss of macroinvertebrate taxa [20,21,78,79] and decreased their overall abundance [80]. In the case of daily downstream fluctuations (79% of the reservoirs in this meta-analysis), organisms can burrow in the sediment (*i.e.*, low mobility taxa such as oligochaetes) or follow the water level up and down (*i.e.*, high mobility swimming taxa such as dragonfly nymphs; [20]). Riverine organisms have evolved and developed adaptations to survive flood and drought hydrological dynamics [81], but lentic taxa are not adapted to extreme water level fluctuations, such as winter drawdown in reservoirs (> 2m amplitude; [22]). Because of that, we were expecting a higher impact and effect size of water level fluctuation in reservoir (*i.e.*, winter drawdown) on macroinvertebrates richness than downstream flow regulation but found no difference nor significant effect of impact type moderator.

Study type moderator

Another moderator that could explain heterogeneity in our results was the type of study. An ideal way to analyze the impact of hydropower on richness is to set the reference conditions as richness before impoundment versus impacted conditions as richness after impoundment, within the same ecosystem (*e.g.*, a reference river that was impounded into an impacted reservoir; longitudinal in time, also known as BACI; [32]). However, studies using this type of methodology are rare, even more so for macroinvertebrates, and such studies were mostly absent in the results from the literature review and if present, did not fulfill the required criteria to be included in the meta-analysis. Here, we accounted for two types of study methodologies; a cross-sectional methodology (*i.e.*, comparing a hydropower impacted ecosystem with a natural/reference ecosystem; 33%) and

longitudinal gradient in space methodology (*i.e.*, upstream of a dam versus downstream of that same dam, within a single ecosystem; 67%), which are both considered as simple CI studies [32]. These methodologies are not ideal, compared to the longitudinal in time methodology (*i.e.*, BACI or BA studies), as their reference spatial point differs from the impacted spatial point, thus introducing some environmental noise and there is no way to control for environmental stochasticity [32]. In the cross-sectional type, studies included inter-ecosystem's variation (*i.e.*, impacted ecosystem was spatially independent from reference/non-impacted ecosystem). This inherently added some heterogeneity in the responses and the impacts could be more difficult to detect. In the longitudinal in space studies (reference upstream and impacted downstream of the dam, at a single point in time), the observations were from the same ecosystem. Here, there is less heterogeneity and thus, we could have expected the results to be less variable. ~~However,~~ there was no significant difference between the two study types in this meta-analysis. Even though the patterns were not significant, we observed a visual negative trend in the spatially longitudinal gradient studies (*i.e.*, higher richness in reference ecosystems, that is upstream of the dam), with a slightly tighter range of variation (not statistically supported). The cross-sectional studies had a tendency toward higher richness in reservoirs, with a larger range of variation. This might highlight a problem with general study design and the choice of the reference ecosystem, and caution is in order when interpreting these trends. Nevertheless, we believe that cross-sectional and longitudinal in space references are the best benchmark available to overcome current limitations regarding the lack of richness data before impoundment in the literature.

Sampling season and gear moderators

We, initially thought we would observe an effect of sampling season when comparing effect sizes since diversity and abundance is known to fluctuate yearly [33] but there was no statistically significant effect of any season that could modulate the meta-analysis outcome. Similarly, we though that depending on the sampling gear we would observe varying diversity because different stratum would be sampled, thus representing different communities of macroinvertebrates [27] but no additional amount of heterogeneity was explained by the sampling gear moderator. The unbalanced nature of the sample sizes could be one of the reasons why these moderators do not provide statistically significant inferences.

The analysis of moderators did not allow a better understanding of the large heterogeneity in the effect sizes and suggests that maybe other moderators, which were not available for the studies included in this meta-analysis, could help tease out some of that heterogeneity. For instance, given that macroinvertebrate are very adapted to their localized environmental conditions (*e.g.*, temperature, granulometry, wave disturbance and macrophytes; [34]), their richness maybe regulated at a much finer scale. Thus, these micro-habitat moderators could be especially relevant to include in a future meta-analysis, although very hard to collect in such a global consolidating endeavour.

Conclusion

This meta-analysis suggested that there is a clear, directional, statistically significant conclusion regarding whether or not hydropower impacts macroinvertebrate richness; macroinvertebrate richness in hydropower, dams and reservoirs impacted environments is significantly lower than in unimpacted environments. However, we also observed a large range of effect sizes, from very negative to very positive impacts of

hydropower. The environmental and methodological heterogeneity in the studies might have hindered the detection of a stronger significant effect, unfortunately none of our moderators helped untangle that heterogeneity. This advises that other moderators, not included in this study due to unavailability among the studies, may be responsible for some of that heterogeneity. We advocate that local, smaller-scale variables pertaining to habitat physicochemical characteristics may bring some clarity about the large heterogeneity in effect sizes. As new studies evaluating the impacts of hydropower on macroinvertebrate richness accumulate, we would recommend that information regarding local habitat variables be available so they could be recorded and evaluated as moderators in future meta-analyses. This meta-analysis was able to highlight a clear directional effect of hydropower on macroinvertebrate richness. As richness is only one aspect of biodiversity, it would be interesting, in future studies, to conduct additional analyses of community composition and functional diversity and thus, get a better portrait of the impact of hydropower on macroinvertebrate biodiversity, not only richness. Moreover, as new studies are available, it would be interesting to keep enriching this meta-analysis to see if the results statistical confidence could strengthen and become even more assertive.

Acknowledgements

We thank the CIRAIG – Polytechnique Montréal for covering the publication fees. We also thank the CSBQ for offering systematic reviews and meta-analyses workshops, which were more than useful for putting together this meta-analysis research article.

References

[1] Strayer DL, Dudgeon D. Freshwater biodiversity conservation: recent progress and future challenges. J North Am Benthol Soc 2010;29:344–58. https://doi.org/10.1899/08-171.1.

[2] Vörösmarty CJ, McIntyre PB, Gessner MO, Dudgeon D, Prusevich A, Green P, et al. Global threats to human water security and river biodiversity. Nature 2010;467:555–61. https://doi.org/10.1038/nature09440.

[3] Dudgeon D, Arthington AH, Gessner MO, Kawabata Z-I, Knowler DJ, Lévêque C, et al. Freshwater biodiversity: importance, threats, status and conservation challenges. Biol Rev 2006;81:163–82. https://doi.org/10.1017/S1464793105006950.

[4] Gracey EO, Verones F. Impacts from hydropower production on biodiversity in an LCA framework—review and recommendations. Int J Life Cycle Assess 2016;21:412–28. https://doi.org/10.1007/s11367-016-1039-3.

[5] Ward JV, Stanford JA. Ecological connectivity in alluvial river ecosystems and its disruption by flow regulation. Regul Rivers Res Manag 1995;11:105–19. https://doi.org/10.1002/rrr.3450110109.

[6] Kummu M, Varis O. Sediment-related impacts due to upstream reservoir trapping, the Lower Mekong River. Geomorphology 2007;85:275–93. https://doi.org/10.1016/j.geomorph.2006.03.024.

[7] Nilsson C, Brown RL, Jansson R, Merritt DM. The role of hydrochory in structuring riparian and wetland vegetation. Biol Rev 2010;85:837–58. https://doi.org/10.1111/j.1469-185X.2010.00129.x.

[8] Poff NL, Allan JD, Bain MB, Karr JR, Prestegaard KL, Richter BD, et al. The Natural Flow Regime. BioScience 1997;47:769–84. https://doi.org/10.2307/1313099.

[9] Bunn SE, Arthington AH. Basic Principles and Ecological Consequences of Altered Flow Regimes for Aquatic Biodiversity. Environ Manage 2002;30:492–507. https://doi.org/10.1007/s00267-002-2737-0.

[10] Friedl G, Wüest A. Disrupting biogeochemical cycles - Consequences of damming. Aquat Sci 2002;64:55–65. https://doi.org/10.1007/s00027-002-8054-0.

[11] Santucci VJ, Gephard SR, Pescitelli SM. Effects of Multiple Low-Head Dams on Fish, Macroinvertebrates, Habitat, and Water Quality in the Fox River, Illinois. North Am J Fish Manag 2005;25:975–92. https://doi.org/10.1577/M03-216.1.

[12] Poff NL, Olden JD, Merritt DM, Pepin DM. Homogenization of regional river dynamics by dams and global biodiversity implications. Proc Natl Acad Sci 2007;104:5732–7. https://doi.org/10.1073/pnas.0609812104.

[13] Liermann CR, Nilsson C, Robertson J, Ng RY. Implications of Dam Obstruction for Global Freshwater Fish Diversity. BioScience 2012;62:539–48. https://doi.org/10.1525/bio.2012.62.6.5.

[14] Jackson HM, Gibbins CN, Soulsby C. Role of discharge and temperature variation in determining invertebrate community structure in a regulated river. River Res Appl 2007;23:651–69. https://doi.org/10.1002/rra.1006.

[15] Takao A, Kawaguchi Y, Minagawa T, Kayaba Y, Morimoto Y. The relationships between benthic macroinvertebrates and biotic and abiotic environmental characteristics downstream of the Yahagi Dam, Central Japan, and the State Change Caused by inflow

from a Tributary. River Res Appl 2008;24:580–97. https://doi.org/10.1002/rra.1135.

[16] Kullasoot S, Intrarasattayapong P, Phalaraksh C. Use of benthic macroinvertebrates as bioindicators of anthropogenic impacts on water quality of Mae Klong river, Western Thailand. Chiang Mai J Sci 2017;44:1356–66.

[17] Kraft KJ. Effect of Increased Winter Drawdown on Benthic Macroinvertebrates in Namakan Reservoir, Voyeurs National Park. Houghton, MI, USA: Michigan Techonological University; 1988.

[18] Englund G, Malmqvist B. Effects of Flow Regulation, Habitat Area and Isolation on the Macroinvertebrate Fauna of Rapids in North Swedish Rivers. Regul Rivers Res Manag 1996;12:433–45. https://doi.org/10.1002/(SICI)1099-1646(199607)12:4/5<433::AID-RRR415>3.0.CO;2-6.

[19] Malmqvist B, Englund G. Effects of hydropower-induced flow perturbations on mayfly (Ephemeroptera) richness and abundance in north Swedish river rapids. Hydrobiologia 1996;341:145–58. https://doi.org/10.1007/BF00018118.

[20] Valdovinos C, Moya C, Olmos V, Parra O, Karrasch B, Buettner O. The importance of water-level fluctuation for the conservation of shallow water benthic macroinvertebrates: an example in the Andean zone of Chile. Biodivers Conserv 2007;16:3095–109. https://doi.org/10.1007/s10531-007-9165-7.

[21] Aroviita J, Hämäläinen H. The impact of water-level regulation on littoral macroinvertebrate assemblages in boreal lakes. Hydrobiologia 2008;613:45–56. https://doi.org/10.1007/s10750-008-9471-4.

[22] White MS, Xenopoulos MA, Metcalfe RA, Somers KM. Water level thresholds of benthic macroinvertebrate richness, structure, and function of boreal lake stony littoral habitats. Can J Fish Aquat Sci 2011;68:1695–704. https://doi.org/10.1139/f2011-094.

[23] Głowacki Ł, Grzybkowska M, Dukowska M, Penczak T. Effects of damming a large lowland river on chironomids and fish assessed with the (multiplicative partitioning of) true/Hill biodiversity measure. River Res Appl 2011;27:612–29. https://doi.org/10.1002/rra.1380.

[24] Serafini Floss EC, Secretti E, Kotzian CB, Spies MR, Pires MM. Spatial and temporal distribution of non-biting midge larvae assemblages in streams in a mountainous region in southern Brazil. J INSECT Sci 2013;13.

[25] Smokorowski KE, Metcalfe RA, Finucan SD, Jones N, Marty J, Power M, et al. Ecosystem level assessment of environmentally based flow restrictions for maintaining ecosystem integrity: a comparison of a modified peaking versus unaltered river. Ecohydrology 2011;4:791–806. https://doi.org/10.1002/eco.167.

[26] Marchetti MP, Esteban E, Smith ANH, Pickard D, Richards AB, Slusark J. Measuring the ecological impact of long-term flow disturbance on the macroinvertebrate community in a large Mediterranean climate river. J Freshw Ecol 2011;26:459–80. https://doi.org/10.1080/02705060.2011.577974.

[27] De Palma A, Sanchez-Ortiz K, Martin PA, Chadwick A, Gilbert G, Bates AE, et al. Chapter Four - Challenges With Inferring How Land-Use Affects Terrestrial Biodiversity: Study Design, Time, Space and Synthesis. In: Bohan DA, Dumbrell AJ, Woodward G, Jackson M, editors. Adv. Ecol. Res., vol. 58, Academic Press; 2018, p. 163–99. https://doi.org/10.1016/bs.aecr.2017.12.004.

[28] Viechtbauer W. Conducting Meta-Analyses in with the metafor Package. J Stat Softw 2010;36:1–48. https://doi.org/10.18637/jss.v036.i03.

[29] Gibson L, Lee TM, Koh LP, Brook BW, Gardner TA, Barlow J, et al. Primary forests are irreplaceable for sustaining tropical biodiversity. Nature 2011;478:378–81. https://doi.org/10.1038/nature10425.

[30] Willig MR, Kaufman DM, Stevens RD. Latitudinal Gradients of Biodiversity: Pattern, Process, Scale, and Synthesis. Annu Rev Ecol Evol Syst 2003;34:273–309. https://doi.org/10.1146/annurev.ecolsys.34.012103.144032.

[31] Rosenzweig ML. Species diversity in space and time. Cambridge, United Kingdom: Cambridge University Press; 1995.

[32] Christie AP, Amano T, Martin PA, Shackelford GE, Simmons BI, Sutherland WJ. Simple study designs in ecology produce inaccurate estimates of biodiversity responses. J Appl Ecol 2019;56:2742–54. https://doi.org/10.1111/1365-2664.13499.

[33] Hill MJ, Sayer CD, Wood PJ. When is the best time to sample aquatic macroinvertebrates in ponds for biodiversity assessment? Environ Monit Assess 2016;188:188–94. https://doi.org/10.1007/s10661-016-5178-6.

[34] McCafferty WP. Aquatic Entomology: The Fishermen's and Ecologists' Illustrated Guide to Insects and Their Relatives. Sudbury, MA, USA: Jones and Bartlett Publishing; 1983.

[35] Wetzel RG. Limnology: Lake and river ecosystems. Third Edition. San Diego, CA, USA: Academic Press; 2001.

[36] Koricheva J, Gurevitch J, Mengersen K, editors. Handbook of Meta-Analysis in Ecology and Evolution. Princeton, NJ, USA: Princeton University Press; 2013.

[37] Moher D. Preferred Reporting Items for Systematic Reviews and Meta-Analyses: The PRISMA Statement. Ann Intern Med 2009;151:264. https://doi.org/10.7326/0003-4819-151-4-200908180-00135.

[38] Clarivate Analytics. Browse, search, and explore journals indexed in the Web of Science. Web Sci Group Clarivate Anal Co 2019. https://mjl.clarivate.com/ (accessed November 27, 2019).

[39] Aroviita J, Hämäläinen H. The impact of water-level regulation on littoral macroinvertebrate assemblages in boreal lakes. Hydrobiologia 2008;613:45–56. https://doi.org/10.1007/s10750-008-9471-4.

[40] Molozzi J, Hepp LU, Callisto M. The additive partitioning of macroinvertebrate diversity in tropical reservoirs. Mar Freshw Res 2013;64:609–17. https://doi.org/10.1071/MF12354.

[41] Mellado-Diaz A, Sanchez-Gonzalez JR, Guareschi S, Magdaleno F, Velasco MT. Exploring longitudinal trends and recovery gradients in macroinvertebrate communities and biomonitoring tools along regulated rivers. Sci Total Environ 2019;695:133774. https://doi.org/10.1016/j.scitotenv.2019.133774.

[42] Bruno D, Belmar O, Maire A, Morel A, Dumont B, Datry T. Structural and functional responses of invertebrate communities to climate change and flow regulation in alpine catchments. Glob Change Biol 2019;25:1612–28. https://doi.org/10.1111/gcb.14581.

[43] Milner VS, Yarnell SM, Peek RA. The ecological importance of unregulated tributaries to macroinvertebrate diversity and community composition in a regulated river. Hydrobiologia 2019;829:291–305. https://doi.org/10.1007/s10750-018-3840-4.

[44] Steel AE, Peek RA, Lusardi RA, Yarnell SM. Associating metrics of hydrologic variability with benthic macroinvertebrate communities in regulated and unregulated

snowmelt-dominated rivers. Freshw Biol 2018;63:844–58. https://doi.org/10.1111/fwb.12994.

[45] Schneider SC, Petrin Z. Effects of flow regime on benthic algae and macroinvertebrates - A comparison between regulated and unregulated rivers. Sci Total Environ 2017;579:1059–72. https://doi.org/10.1016/j.scitotenv.2016.11.060.

[46] Vaikasas S, Palaima K, Pliūraite V. Influence of hydropower dams on the state of macroinvertebrates assemblages in the Virvyte river, Lithuania. J Environ Eng Landsc Manag 2013;21:305–15. https://doi.org/10.3846/16486897.2013.796956.

[47] Dolédec S, Simon L, Blemus J, Rigal A, Robin J, Mermillod-Blondin F. Multiple stressors shape invertebrate assemblages and reduce their trophic niche: A case study in a regulated stream. Sci Total Environ 2021;773:145061. https://doi.org/10.1016/j.scitotenv.2021.145061.

[48] Wang H, Chen Y, Liu Z, Zhu D. Effects of the “Run-of-River” Hydro Scheme on Macroinvertebrate Communities and Habitat Conditions in a Mountain River of Northeastern China. Water 2016;8:31. https://doi.org/10.3390/w8010031.

[49] Nukazawa K, Shirasaka K, Kajiwara S, Saito T, Irie M, Suzuki Y. Gradients of flow regulation shape community structures of stream fishes and insects within a catchment subject to typhoon events. Sci Total Environ 2020;748:141398. https://doi.org/10.1016/j.scitotenv.2020.141398.

[50] Quadroni S, Salmaso F, Gentili G, Crosa G, Espa P. Response of benthic macroinvertebrates to different hydropower off-stream diversion schemes. Ecohydrology 2020;n/a:e2267. https://doi.org/10.1002/eco.2267.

[51] Vilenica M, Pozojević I, Vučković N, Mihaljević Z. How suitable are man-made water bodies as habitats for Odonata? Knowl Manag Aquat Ecosyst 2020:13. https://doi.org/10.1051/kmae/2020008.

[52] Uieda VS, Marçal SF. How the structure if a phytophilous chironomid assemblage responds to a lake level drawdown for submerged macropohyte control in a tropical reservoir. Limnetica 2020;39:555–69.

[53] Cazaubon A, Giudicelli J. Impact of the residual flow on the physical characteristics and benthic community (algae, invertebrates) of a regulated Mediterranean river: the Durance, France. Regul Rivers Res Manag 1999;15:441–61. https://doi.org/10.1002/(SICI)1099-1646(199909/10)15:5<441::AID-RRR558>3.0.CO;2-9.

[54] Olson DM, Dinerstein E, Wikramanayake ED, Burgess ND, Powell GVN, Underwood EC, et al. Terrestrial Ecoregions of the World: A New Map of Life on EarthA new global map of terrestrial ecoregions provides an innovative tool for conserving biodiversity. BioScience 2001;51:933–8. https://doi.org/10.1641/0006-3568(2001)051[0933:TEOTWA]2.0.CO;2.

[55] Mitchell M, Muftakhidinov B, Winchen T. Engauge Digitizer - Extract data points from images of graphs 2017. http://markummitchell.github.io/engauge-digitizer (accessed March 3, 2012).

[56] Borenstein M, Hedges LV, Higgins JPT, Rothstein HR. Introduction to Meta-Analysis. United Kingdom: John Wiley & Sons; 2011.

[57] Hedges LV. Distribution Theory for Glass’s Estimator of Effect size and Related Estimators. J Educ Stat 1981;6:107–28. https://doi.org/10.3102/10769986006002107.

[58] Wickham H. ggplot2: Elegant Graphics for Data Analysis. Springer-Verlag New

York; 2016.
[59] Sterne JAC, Egger M. Funnel plots for detecting bias in meta-analysis: Guidelines on choice of axis. J Clin Epidemiol 2001;54:1046–55. https://doi.org/10.1016/S0895-4356(01)00377-8.
[60] Sterne JAC, Egger M. Regression methods to detect publication and other bias in meta-analysis. In: Rothstein HR, Sutton AJ, Borenstein M, editors. Publ. Bias Meta-Anal. Prev. Assess. Adjust. First Edition, Chichester, United Kingdom: Wiley; 2005, p. 99–110.
[61] Huedo-Medina TB, Sánchez-Meca J, Marín-Martínez F, Botella J. Assessing heterogeneity in meta-analysis: Q statistic or I^2 index? Psychol Methods 2006;11:193–206. https://doi.org/10.1037/1082-989X.11.2.193.
[62] Moeyaert M, Ugille M, Beretvas SN, Ferron J, Bunuan R, Noortgate WV den. Methods for dealing with multiple outcomes in meta-analysis: a comparison between averaging effect sizes, robust variance estimation and multilevel meta-analysis. Int J Soc Res Methodol 2017;20:559–72. https://doi.org/10.1080/13645579.2016.1252189.
[63] Fisher Z, Tipton E. robumeta: an R package for robust variance estimation in meta-analysis 2017.
[64] R Core Team. R: a language and environment for statistical computing. Vienna, Austria: R Foundation for Statistical Computing; 2021.
[65] van Emden HF. Statistics for Terrified Biologists. First Edition. Oxford, United Kingdom: Wiley-Blackwell; 2008.
[66] Spellman FR, Whiting NE. Handbook of Mathematics and Statistics for the Environment. First Edition. Boca Raton, FL, USA: CRC Press; 2013.
[67] Hillebrand H. On the Generality of the Latitudinal Diversity Gradient. Am Nat 2004;163:192–211. https://doi.org/10.1086/381004.
[68] Lomolino MV, Riddle BR, Brown JH. Biogeography. 3rd ed. Sunderland, MA, USA: Sinauer Associates; 2006.
[69] Turgeon K, Turpin C, Gregory- Eaves I. Dams have varying impacts on fish communities across latitudes: a quantitative synthesis. Ecol Lett 2019;22:1501–16. https://doi.org/10.1111/ele.13283.
[70] Covich AP. Geographical and Historical Comparisons of Neotropical Streams: Biotic Diversity and Detrital Processing in Highly Variable Habitats. J North Am Benthol Soc 1988;7:361–86. https://doi.org/10.2307/1467297.
[71] Vinson MR, Hawkins CP. Broad-scale geographical patterns in local stream insect genera richness. Ecography 2003;26:751–67. https://doi.org/10.1111/j.0906-7590.2003.03397.x.
[72] Allan JD, Castillo MM. Stream Ecology: Structure and Function of Running Waters. Second Edition. The Netherlands: Springer Science & Business Media; 2007.
[73] Pearson RG, Boyero L. Gradients in regional diversity of freshwater taxa. J North Am Benthol Soc 2009;28:504–14. https://doi.org/10.1899/08-118.1.
[74] Scott RW, Barton DR, Evans MS, Keating JJ. Latitudinal gradients and local control of aquatic insect richness in a large river system in northern Canada. J North Am Benthol Soc 2011;30:621–34. https://doi.org/10.1899/10-112.1.
[75] Vinson MR, Hawkins CP. Effects of Sampling Area and Subsampling Procedure on Comparisons of Taxa Richness among Streams. J North Am Benthol Soc 1996;15:392–9. https://doi.org/10.2307/1467286.
[76] Heino J. Biodiversity of Aquatic Insects: Spatial Gradients and Environmental

Correlates of Assemblage-Level Measures at Large Scales. Freshw Rev 2009;2:1–29. https://doi.org/10.1608/FRJ-2.1.1.
[77] Shah DN, Domisch S, Pauls SU, Haase P, Jähnig SC. Current and future latitudinal gradients in stream macroinvertebrate richness across North America. Freshw Sci 2014;33:1136–47. https://doi.org/10.1086/678492.
[78] Palomäki R, Koskenniemi E. Effects of bottom freezing on macrozoobenthos in the regulated lake Pyhäjärvi. Arch Für Hydrobiol 1993;128:73–90.
[79] Palomäki R. Response by macrozoobenthos biomass to water level regulation in some Finnish lake littoral zones. Hydrobiologia 1994;286:17–26. https://doi.org/10.1007/BF00007277.
[80] Trottier G, Embke H, Turgeon K, Solomon C, Nozais C, Gregory-Eaves I. Macroinvertebrate abundance is lower in temperate reservoirs with higher winter drawdown. Hydrobiologia 2019;834:199–211. https://doi.org/10.1007/s10750-019-3922-y.
[81] Humphries P, Baldwin DS. Drought and aquatic ecosystems: an introduction. Freshw Biol 2003;48:1141–6. https://doi.org/10.1046/j.1365-2427.2003.01092.x.

Supporting information

S1 Table. PRISMA checklist. Specific to this meta-analysis, from Moher et al. [37].

S1 Fig. Funnel plot for this meta-analysis. No statistically significant asymmetry is observed (z = -1.74, P = 0.08).

S2 Table. Metadata table showing all variables for each study included in this meta-analysis [14-18, 20, 22, 25-26, 40-54]. Use S3 Table as a companion table to get more information on each variable.

S3 Table. Companion table describing all variables in S2 Table.

S4 Table. Table showing the sensitive analysis outputs. Rho values (ρ) ranges from 0 to 1 and mean effect size (ES), standard error (SE) and between study variance (τ^2) estimates are relatively insensitive to these varying ρ values.

Figure1

Click here to access/download;Figure;Fig1.tiff

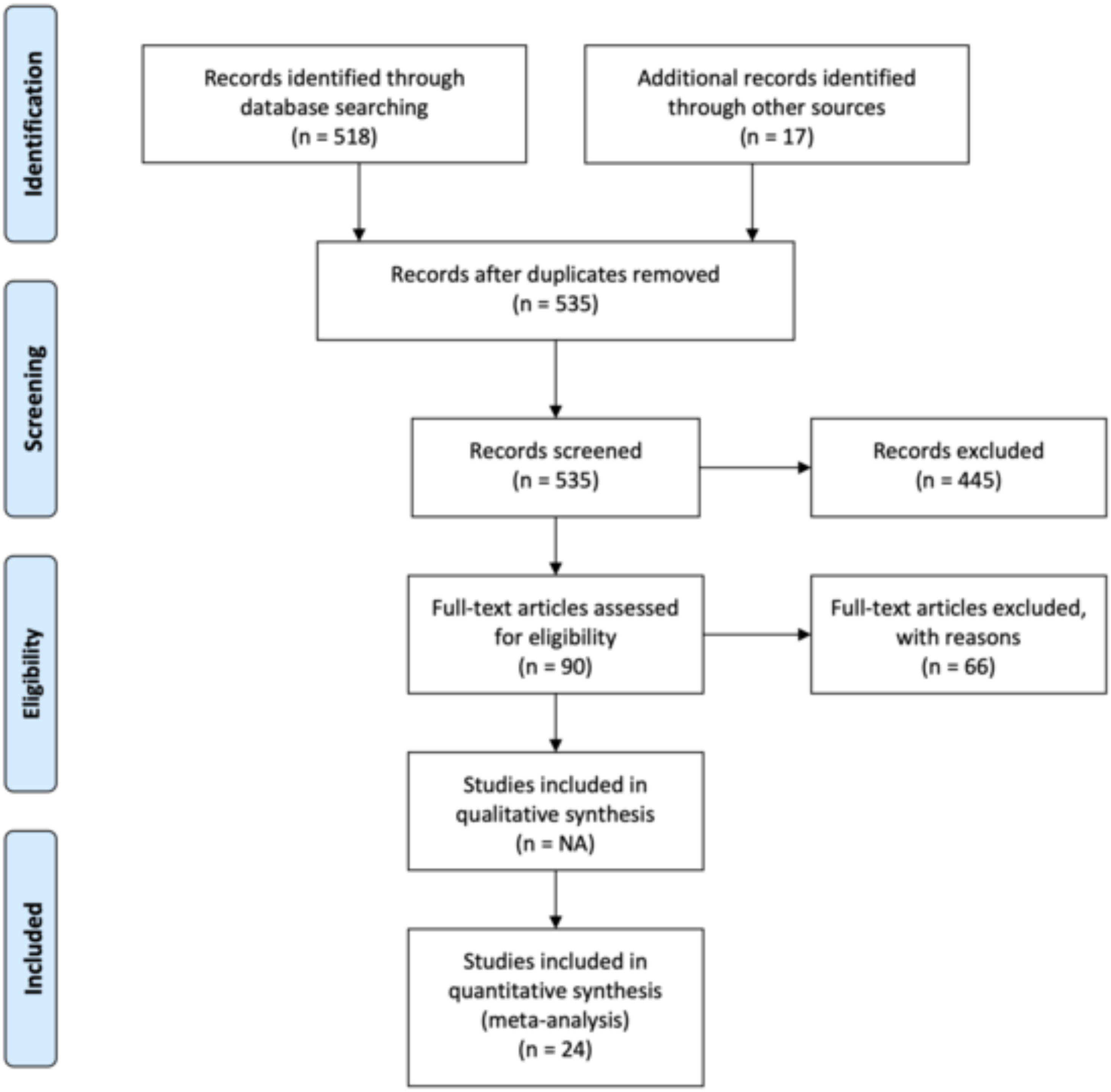

Figure2 Click here to access/download;Figure;Fig2.tiff

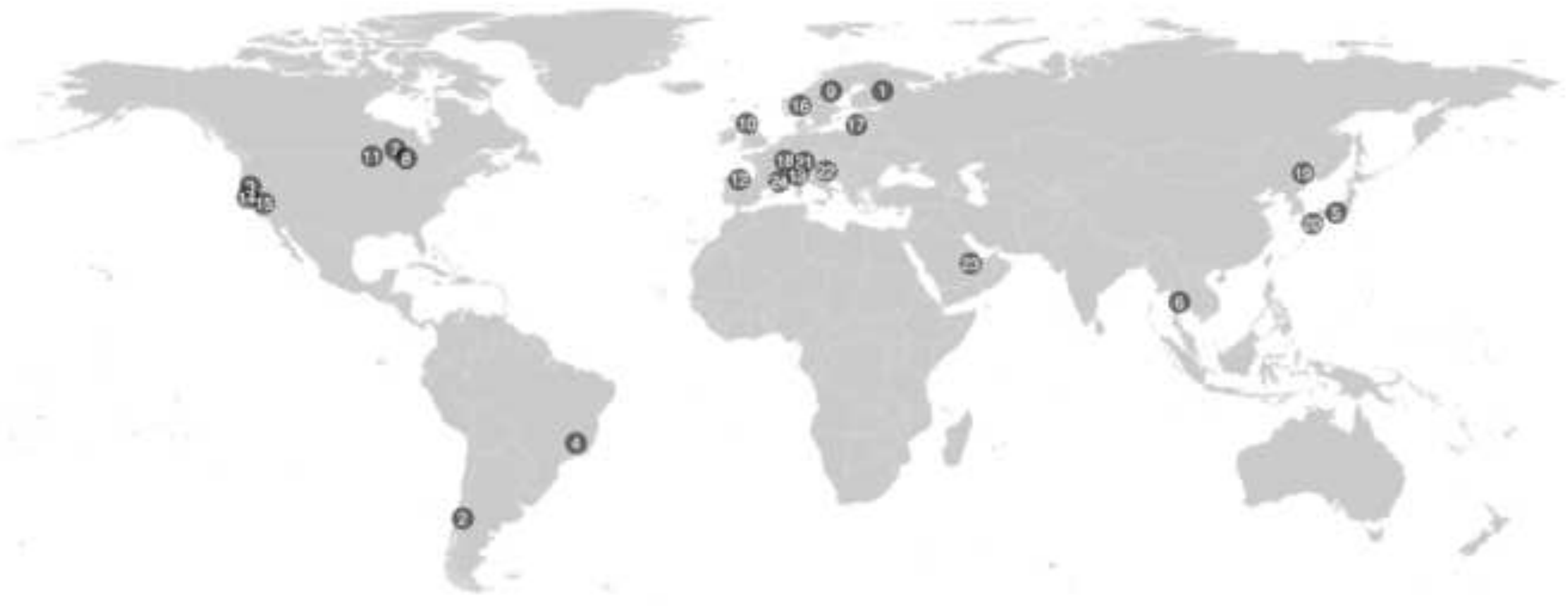

Figure3

Click here to access/download;Figure;Fig3.tiff

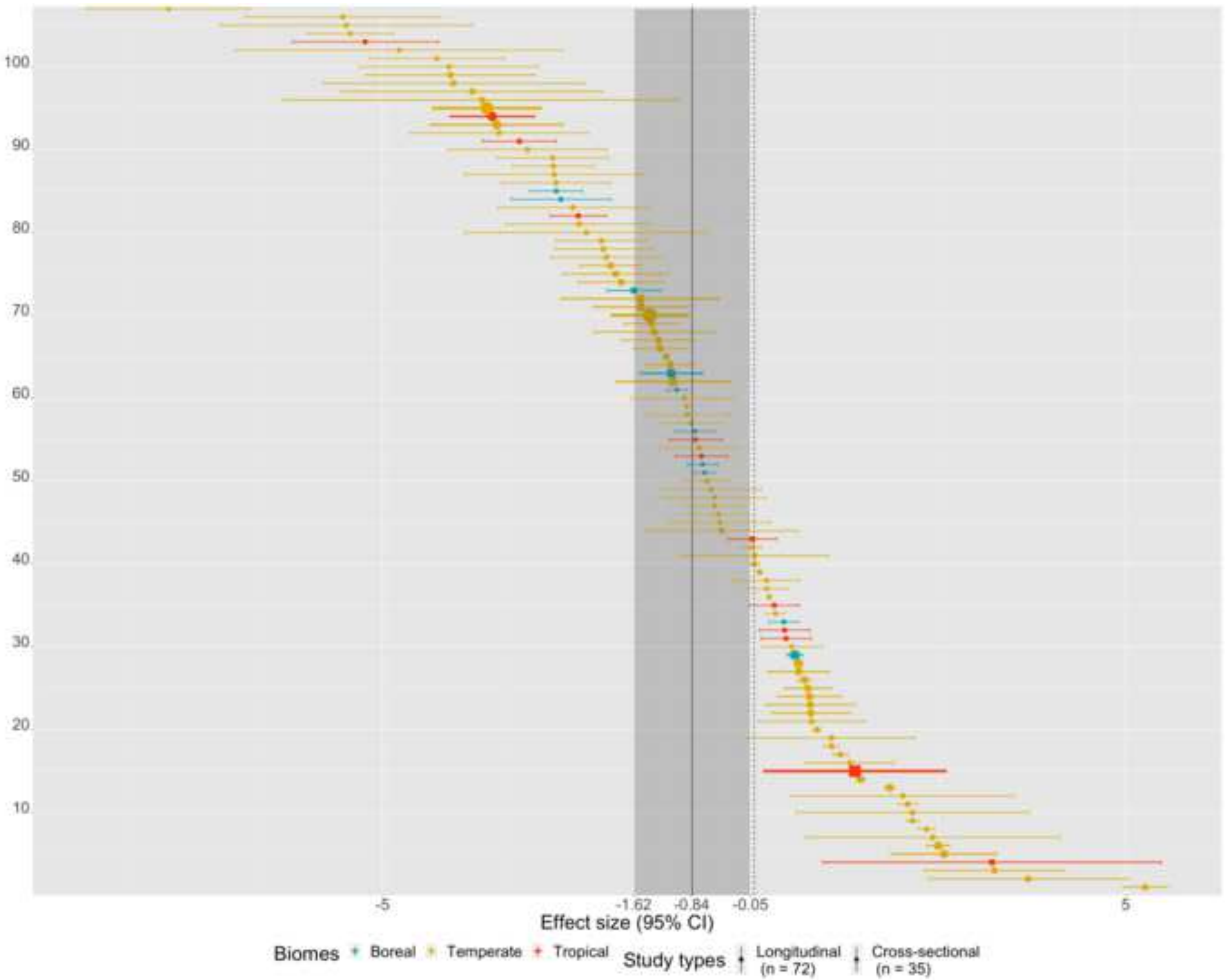

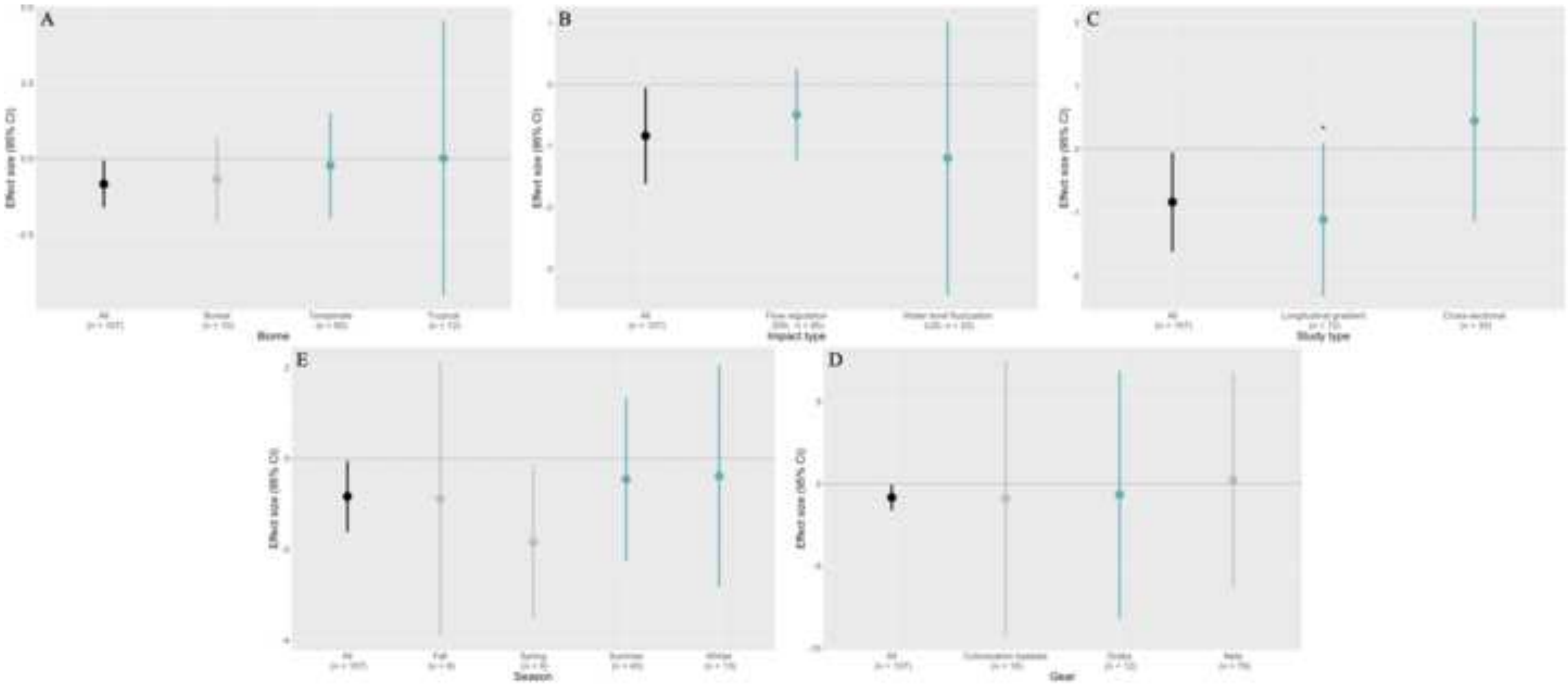
A
B
C
E
D
Effect size (95% CI)
Biome
Impact type
Study type
Season
Gear

Click here to access/download
Supporting Information
Manuscript_Trottier.etal_SUPPORTING.INFORMATION.docx

~~Title:~~ The impacts of hydropower on freshwater macroinvertebrate richness: A global meta-analysis

Formatted: Centered

Gabrielle Trottier[1*], Katrine Turgeon[2], Daniel Boisclair[3], Cécile Bulle[4] and Manuele Margni[1,5]

[1] CIRAIG, Département de mathématique et génie industriel, Polytechnique Montréal, Montréal, Québec, Canada
*Corresponding author: gabrielle.trottier@polymtl.ca, 514-340-4711 #2136

[2] ISFORT, Université du Québec en Outaouais, Ripon, Québec, Canada
katrine.turgeon@uqo.ca

[3] Département de sciences biologiques, Université de Montréal, Montréal, Québec, Canada
daniel.boisclair@umontreal.ca

[4] CIRAIG, Département de stratégie, responsabilité sociale et environnementale, École des sciences de la gestion, Université du Québec à Montréal, Montréal, Québec, Canada
bulle.cecile@uqam.ca

[5] Institute of Sustainable Energy, HES-SO Valais-Wallis, Sion, Switzerland
manuele.margni@polymtl.ca

Field Code Changed

Abstract

Hydroelectric dams and their reservoirs have been suggested to affect freshwater biodiversity. However, studies investigating the consequences of hydroelectric dams and reservoirs on macroinvertebrate richness have reached opposite conclusions. We carried out a meta-analysis devised to elucidate the effects of hydropower, dams and reservoirs on macroinvertebrate richness while accounting for the potential role played by moderators such as biomes, impact types, study designs, sampling seasons and gears. We used a random/~~ and ~~mixed-~~ ~~effects model, combined with robust variance estimation, to conduct the meta-analysis on 107 pairs of observations (*i.e.*, impacted versus reference) extracted from 24 studies (more than one observation per study). Hydropower, dams and reservoirs did significantly impact macroinvertebrate richness in a clear, directional and statistically significant way, where macroinvertebrate richness in hydropower, dams and reservoirs impacted environments were significantly lower than in unimpacted environments. However, we also observed a large range of effect sizes, from very negative to very positive impacts of hydropower. We tried to account for the large variability in effect sizes using moderators, but none of the moderators included in the meta-analysis had statistically significant effects. This suggests that some other moderators (unavailable for the 24 studies) might be important (*e.g.*, temperature, granulometry, wave disturbance and macrophytes) and that macroinvertebrate richness may be driven by local, smaller scale processes. As new studies become available, it would be interesting to keep enriching this meta-analysis, as well as collecting local habitat variables, to see if we could statistically strengthen and deepen the conclusions of this meta-analysis.

Introduction

Freshwater ecosystems are vital resources for humans and support a biota that is rich, sensitive and characterized by a high level of endemicity [1]. Ecosystems functions and integrity often depend on biodiversity, which can be described by three indices: species richness (*i.e.*, number of species), community assemblage (*i.e.*, proportions of different species or taxonomic groups in the community) and functional diversity (*i.e.*, variability in organisms' traits that can influence ecosystem functioning; [1]). For millennia, humans have used freshwater ecosystems, through water extraction for drinking and irrigation purposes, water regulation for hydropower production, flood control and recreation [1], but these usages often come with a cost on freshwater ecosystems biodiversity [2,3].

Hydroelectric dams and the creation of reservoirs, at all stages of their life cycle (*i.e.*, from the construction, to operation and decommission of a dam), can affect freshwater biodiversity [4]. Dams create a physical barrier, which can impair the natural flow of water, sediments and nutrients [5,6] and limit the movement of organisms [7]. The alteration of the natural hydrological regime can affect freshwater biodiversity through various biological mechanisms (*i.e.*, mortality through desiccation, mismatch timing in life history strategies, lotic to lentic community changes, reduction/extirpation of endemic and specialist species; [8,9]) and through degraded water quality (anoxic or hypoxic releases [dissolved oxygen], hypolimnetic or epilimnetic releases [temperature], pH, organic carbon, turbidity; [4,10–13]).

Studies investigating the impact of hydropower on macroinvertebrate richness drew contrasting conclusions. Some studies reported that macroinvertebrate richness is negatively impacted by hydropower, through general flow regulation [14–16] and water

level fluctuation (or drawdown; [17–22]). Others observed higher macroinvertebrate richness downstream of a dam [23,24] or in regulated rivers (as opposed to natural ones; [25]). Finally, Marchetti et al. [26] found little difference between impacted flows (*i.e.*, dam-induced permanent low flow) and "natural-like" flows (*i.e.*, high flows in winter and spring, low flows in summer and falls). A meta-analysis could ~~allow to~~ elucidate ~~the~~ patterns and interactions that may exist between hydropower, macroinvertebrate richness, and the context in which studies have been conducted.

Many challenges can be encountered when conducting a meta-analysis, and many ecological facets of studied ecosystems can influence the magnitude and significance of human impacts [27], along with study-specific methodological characteristics (*e.g.*, different study design). The influence and the variability brought about by these facets and characteristics can be accounted for through variables, also called moderators in meta-analysis [28]. For instance, the location of each study site can influence the observed effects [27,29,30]. A latitudinal biodiversity gradient is a good example of the influence of spatial location. Species richness is known to be highest in the tropics and lowest at the poles [30,31]. Thus, losing few species to hydropower activities will not have the same repercussions on low diversity macroinvertebrate communities than higher diversity communities (*e.g.*, losing 2/10 species [20% loss] versus 2/25 species [8% loss]).~~.~~ Hydropower can lead to different types of impacts. A study can analyze the impacts of hydropower upstream of a dam, that is in the reservoir, or downstream of a dam. Impacts also varies depending on the type of water management in place, storage reservoir with winter water level drawdown, hydropeaking or typical run-of-river hydrological regime. These variations in studies, along with the location under study, can introduce variability

and heterogeneity in the results, which can be accounted for through moderators. The experimental design can also influence how human-induced impacts magnitude are reflected in a study [27]. As demonstrated in Christie et al. [32], different sampling designs may affect the conclusion of a study. Using simulations, they demonstrated that Before-After (BA), Control-Impact (CI; analogous to space-for-time substitution) and After designs are far less accurate than Randomized Controlled Trials (RCT) and Before-After Control-Impact (BACI) designs. RCT and BACI are much harder to implement in ecology because true randomization can be difficult with larger scale designs and getting data before the impacts or human intervention is sometimes impossible. Thus, we must account for the effect of the experimental design on a study outcome, especially in a meta-analysis, where the effects of multiple different studies are combined. Sampling season can also influence the results across studies, as macroinvertebrate communities differs in terms of abundance and diversity throughout the year (*i.e.*, maximum diversity in late summer and autumn versus underrepresented diversity in spring and early/mid-summer; [33]). At a more local scale, the habitat stratum that is sampled is also most likely to influence the results [27], especially when studying macroinvertebrates. These organisms possess characteristics that make them highly adapted to their environment [34] and because lakes, reservoirs and river beds are so heterogeneous, macroinvertebrates are often patchily distributed, requiring extensive sampling [35]. Thus, the type of sampling gear used to sample will likely affect the type of organisms inventoried in each study. However, using sampling gear as a proxy for habitat stratum might need a caveat as many biomonitoring protocols (*e.g.*, United State Environmental Protection Agency) use nets for multiple-

habitat assessments. In this study, we assumed that different sampling methods would collect different macroinvertebrate communities.

The objective of this manuscript is to conduct a meta-analysis about the impacts of hydropower dams and their reservoirs on macroinvertebrates richness while accounting for a series of moderators defining the context of the studies included. The moderators included in this manuscript are the following: 1) biomes (*i.e.*, boreal, temperate, and tropical), a proxy for location/latitudinal gradient, 2) type of impact, which is reflected by the position of a sample in relation to the dam (*i.e.*, upstream or downstream of the dam). Downstream stations are impacted by a reduced flow and hydropeaking dynamics, whereas upstream stations are impacted by drawdown and water level fluctuations due to reservoir management, 3) type of study designs such as cross-sectional (*i.e.*, reference natural lake versus impacted reservoir) and longitudinal spatial gradient (*i.e.*, upstream of a dam [reservoir] versus downstream of a dam [river]), which are two different variants of CI study design, 4) sampling seasons (*i.e.*, spring, summer, fall, winter; we were interested in the coarser temporal effect of seasons rather than daily changes in colonization following water regulation) and 5) sampling gears, a proxy for habitat stratum (*i.e.*, grabs and nets). Even if richness constitutes only one of three components of biodiversity, we chose to focus on macroinvertebrate richness because it is easier to quantify and extract from the scientific literature than community composition and also a lot more common than functional diversity.

Commented [GT1]: Should I move it up just before the description of the moderators used in this study?

Methodology

~~A meta-analytic framework was used to assess the impacts of hydropower on macroinvertebrate richness, for different biomes, impact types, study designs, seasons~~

Formatted: Not Highlight

~~and sampling gear. A meta-analysis is a method of research synthesis that is based on expressing results from multiple studies that share a given research subject (but different sampling strategies, sample sizes or sampling gears) on a common comparison scale, and aims to integrate findings, establish generalizations or resolve conflicts [36]. Whereas a research synthesis is usually performed qualitatively, a meta-analysis is a powerful, informative and unbiased tool that allows the quantification of generalizations through various statistical methods [36]. It statistically combines the magnitude and the direction of results or outcomes (later referred to as effect sizes) of multiple studies sharing a common research objective [36]. For example, when comparing richness between an impacted and a natural habitat, the magnitude of the difference in richness (*i.e.*, delta) and its direction (*i.e.*, either gain or loss of taxa) constitute the effect size. Meta-analysis has many advantages over other methods (*i.e.*, narrative review, vote counting). Namely, it takes into account sample size and statistical power from each study (with potentially different sampling strategies), assesses the magnitude and statistical significance of the mean effect size, and allows the analysis of multiple sources of variation among studies (*i.e.*, moderators; [36]). For those reasons, we chose this method over the others.~~

Achieving our objective requires to first establish a research strategy, second to do a data collection comprising information regarding macroinvertebrate richness in hydropower impacted habitat versus reference ones, biome, the type of impact, study design, sampling seasons and gear. Third, it requires to compute effect sizes for each study and finally, combine them to assess if the mean effect size is significantly different from zero [36] and if the presence of other moderators can influence these results.

Research strategy

In this study, we used the PRISMA (Preferred Reporting Items for Systematic and Meta-Analyses) methodology, flow diagram (~~S1~~ Fig 1) and checklist (S1 Table) proposed by Moher et al. [37], to report systematic literature reviews and meta-analyses. A systematic literature review was conducted using the Web of Science Core Collection database, which includes all journals indexed in Science Citation Index Expanded (SCI-EXPANDED), Social Sciences Citation Index (SSCI), Arts & Humanities Citation Index (A&HCI), Conference Proceedings Citation Index – Science (CPCI-S), Conference Proceedings Citation Index – Social Science & Humanities (CPCI-SSH) and Emerging Sources Citation Index (ESCI; [38]). The research strategy was constrained between 1989 (earliest searchable year) and 2021 and contained a combination of the four following field of research: 1) hydropower (hydropower OR hydroelectric* OR dam OR dams OR reservoir* OR impound* OR run-of-river OR "run of river" OR drawdown* OR hydropeak* OR dam* OR "water level fluctuation" OR "water-level fluctuation" OR "water level variation" OR "water-level variation" OR "water level regulation" OR "water-level regulation" OR "water level manipulation" OR "water-level manipulation" OR "water management"), 2) biodiversity (biodiversity OR richness), 3) freshwater ecosystems (freshwater OR aquatic) and 4) aquatic insects (*invertebrate* OR *benth* OR insect* OR arthropod*). Research strategy also excluded all studies pertaining to beaver and agricultural dams (NOT beaver* NOT agricult*). The research strategy resulted in 518 research articles, as per September 1st 2021.

INSERT FIG 1 HERE

Fig 1. PRISMA diagram. Specific to this meta-analysis, from Moher et al. [37].

The results were ~~sorted by Web of Science relevance, which ranks records based on the number of search terms found in each record [39], and~~ extracted as a list to further

evaluate the relevance of every study~~ies~~ based on a list of criteria. The following criteria were applied to assess the inclusion of any study in the meta-analysis: 1) the study had to refer specifically to hydropower related impacts (*i.e.*, reservoir, run-of-the-river or hydropeaking, multi-purpose reservoirs were also checked for hydropower impacts), 2) the scope of the study had to address freshwater ecosystems and macroinvertebrates and 3) the study had to be empirical (*i.e.*, excluding literature reviews, modelling exercises) and provide an explicit richness, error and sample size value for both a reference and impacted site (*i.e.*, cross-sectional studies [reference versus impacted]) or gradient of impact (longitudinal spatial gradient studies [upstream of the dam/reservoir versus one or multiple sites downstream of the dam]). Out of the 518 research studies that resulted from the research strategy, only 24 met the above criteria (see geographical disposition of studies in Fig 2~~1~~).

INSERT FIG 2~~1~~ HERE

Fig 2. World map showing the geographical disposition of the studies used in this meta-analysis.~~;~~ (1) Aroviita and Hämäläinen (2008) *[39]*, (2) Valdovinos et al. (2007) *[20]*, (3) Marchetti et al. (2011) *[26]*, (4) Molozzi et al. (2013) *[40]*, (5) Takao et al. (2008) *[15]*, (6) Kullasoot et al. (2017) *[16]*, (7) White et al. (2011) *[22]*, (8) Smokorowski et al. (2011) *[25]*, (9) Englund and Malmqvist (1996) *[18]*, (10) Jackson et al. (2007) *[14]*, (11) Kraft (1988) *[17]*, (12) Mellado-Dìaz et al. (2019) *[41]*, (13) Bruno et al. (2019) *[42]*, (14) Milner et al. (2019) *[43]*, (15) Steel et al. (2018) *[44]*, (16) Schneider and Petrin (2017) *[45]*, (17) Vaikasas et al. (2013) *[46]*, (18) Doledec et al. (2021) *[47]*, (19) Wang et al. (2016) *[48]*, (20) Nukazawa et al. (2020) *[49]*, (21) Quadroni et al. (2020) *[50]*, (22) Vilenica et al. (2020) *[51]*, (23) Uieda and Marcal (2020) *[52]* and (24) Cazaubon and Giudicelli (1999) *[53]*.

Data collection

Richness metrics and moderators were collected for each of these 24~~17~~ studies by the corresponding author ~~-~~(S2 and S3 Tables). We extracted richness (*i.e.*, number of taxa), error measure (*e.g.*, standard deviation), sample size (*i.e.*, number of reference observations and impacted observations used to compute richness and its associated error) and a suite of moderators such as biome (*i.e.*, boreal, temperate and tropical; [54]), type of impact (*i.e.*, water level fluctuations upstream, due to reservoir management, or flow regulation

downstream due to dam operations), type of study, (*i.e.*, cross-sectional [reference natural lake versus impacted reservoir] or longitudinal spatial gradient [reference; upstream of the dam/reservoir versus impacted; downstream of the dam]), sampling season (*i.e.*, spring [March to May], summer [June to August], fall [September to November] and winter [December to February], according to the hemisphere) and sampling gear (*i.e.*, net, grab or colonization basket). These moderators were chosen based on the availability of said moderators in each of the 24~~17~~ studies, their potential influence on macroinvertebrate richness and complemented with expert judgement. In studies where meaningful data were presented exclusively in graphical format, values were extracted using Engauge Digitizer 10.4 [55].

Effect size

To compute effect sizes, we calculated the standardized mean differences, also called Cohen's *d* – which expresses the distance between two means (*i.e.*, impact and reference) in terms of their common standard deviation [56]. For most studies – except Takao et al. [15], Schneider and Petrin [45] and Vilenica et al. [51], we computed at least two effect sizes per study, leading to a total of 107 effect sizes, with a certain level of within-study dependency (*i.e.*, effect sizes in one study are not entirely independent from each other). Cohen's *d* is calculated as per equation 1 [56]:

$$d = \frac{\bar{X}_1 - \bar{X}_2}{\sqrt{\left(\frac{(n_1 - 1)s_1^2 + (n_2 - 1)s_2^2}{n_1 + n_2 - 2}\right)}} \qquad (1)$$

where $\bar{X}_1$ and $\bar{X}_2$ are the mean richness, s_1^2 and s_2^2 are the standard deviation (SD) and, n_1 and n_2 are the number of observations used to compute the mean and SD for impacted and

reference samples, respectively. The common variance (V_d) associated with the effect size (*d)* is calculated using equation 2:

$$V_d = \frac{n_1 + n_2}{n_1 \cdot n_1} + \frac{d^2}{2(n_1 + n_2)} \tag{2}$$

In the case of smaller sample size (usually < 20 studies), a correction factor is applied to Cohen's *d* to reduce the positive bias (negligible with bigger sample size) and to provide a better estimate. The corrected effect size is then called a Hedges' g [57]. A small sample correction factor (J) was computed using equation 3:

$$J = 1 - \frac{3}{4df - 1} \tag{3}$$

where df refers to the degrees of freedom ($n_1 + n_2 - 2$). Thus, the corrected effect size *g* and variance V_g are calculated following equations 4 and 5, respectively:

$$g = J \cdot d \tag{4}$$

$$V_g = J^2 \cdot V_d \tag{5}$$

A positive g means the impacted environment or sample has more richness in comparison to a reference environment or sample. The *metafor* package [28] was used to compute the effect sizes and sampling variances (*i.e., escalc* function) and the *ggplot2* package [58] was used to graphically visualize the results of the meta-analysis.

Data analysis

Publication bias

Studies with large significant results are more likely to be published than studies with non-significant results, this is called publication bias [36]. A funnel plot was used to evaluate the presence of a publication bias in the meta-analysis [59], and a regression test for funnel plot was used to detect potential asymmetry (*i.e.*, if only large significant studies,

range of outcomes is not well represented and studies with non-significant results might not even be present; [60]). The *metafor* package [28] was used for asymmetry analysis (*i.e.*, *funnel* and *regtest* functions).

Heterogeneity

There are two sources of heterogeneity in a meta-analysis 1) the heterogeneity due to sampling error, or the within-study heterogeneity (*i.e.*, methodological heterogeneity), which is always present in meta-analyses as every study uses different samples, and 2) the true heterogeneity due to s~~S~~pecific study characteristics (*e.g.*, biome) and dissimilarities in methodologies among studies (*e.g.*, study design), which can introduce variability ~~, or heterogeneity (not only due to within study sampling error),~~ among true effect sizes [28,61].The combination of the methodological and the true heterogeneity is referred to as the total heterogeneity. It is interesting to examine this total he~~he~~terogeneity and identify the different moderators and their relative contributions to the magnitude and direction of these effect sizes [36]. We evaluated the statistical significance and the magnitude of the total heterogeneity of effect sizes among studies (*i.e.*, heterogeneity analysis) using~~, respectively,~~ the *Q* statistic, followed by the ~~and~~ I^2 index to identify how much of this total heterogeneity is due to true heterogeneity -[61]. The *metafor* package [28] was used for heterogeneity analysis (*i.e.*, *rma* function).

Random/ ~~and~~ mixed-effects model: dealing with heterogeneity

Two types of meta-analytic models can be used in a meta-analysis, fixed-effects or random/mixed-effects. A fixed-effects model considers only the studies included in the meta-analysis and within study sampling variability, not between studies [36]. No inference can be made outside this set of studies (*i.e.*, conditional inferences; [28]). A random/mixed-

effects model considers the set of analyzed studies as a sample of a larger population of studies [28]. Thus, it allows the researcher to make inferences regarding what would be found if an entire new meta-analysis, with a different set of studies, was performed (*i.e.*, unconditional inferences; [28]). It also allows two sources of variation, within and among studies [36]. Such an approach is especially appropriate when dealing with heterogeneity among studies [61] and, with a random/mixed-effects model approach, it is also possible to include moderators, which can account for some of that heterogeneity [28]. It is important to highlight that these moderators do not impact species richness, they rather explain additional heterogeneity in the meta-analysis effect sizes. Here, a random/~~ and~~ mixed-~~ ~~effects modelling is preferred to a fixed-effects modelling approach since 1) a significant amount of heterogeneity was found in the previous heterogeneity analysis and 2) because it offers the possibility to model and explain some of that heterogeneity using moderators [56].

Robust variance estimation: dealing with dependency

If our effect sizes were all independent from each other, we could have simply used a random/~~ and ~~mixed-~~ ~~effects model. However, because this meta-analysis is dealing with multiple effect sizes per study, where observations are not methodologically and spatially independent from each other, it is inappropriate to use a regular meta-analysis approach (*i.e.*, random/~~ and ~~mixed-~~ ~~effects model), where the effect sizes are assumed to be independent. One way to account for dependency of effect sizes is to combine the random/~~ and ~~mixed-~~ ~~effects model with the robust variance estimation (RVE) method. The RVE estimates the overall effect size over studies using a weighted mean of the observed effect sizes [62]. It doesn't require knowledge about the within-study covariance, it can be applied

to any type of dependency and effect sizes, it simultaneously accommodates for multiple sources of dependencies, it does not require the effect sizes to comply to any particular distribution assumptions, it leads to unbiased fixed-~~-~~effects and standard errors estimates and can also give an estimate of among-study variance [62,63]. Because the most common source of dependence within the effect sizes in this meta-analysis is the correlated nature of the observations (*i.e.*, multiple measures within a study; methodological and spatial correlation) and not the hierarchical nature (*i.e.*, common nesting structure between studies; a sample within a transect, within site and within a lake), a correlated effects weighting method was used [63]. Thus, we used a RVE based on a correlated effects model and adjusted for small sample size (< 40 studies; [63]). Finally, a sensitivity analysis was computed to assess the effect of a varying rho (ρ) value, which is a user-specified value of the within-study effect sizes correlation (*i.e.*, the correlation between two samples taken in the same water body in one specific study – spatial and methodological correlation; [63]). The *robumeta* package [63] was used to fit the RVE meta-regression model (*i.e.*, *robu* function) and to compute the sensitivity analysis (*i.e.*, *sensitivity* function). All statistical analyses were made using R version 3.0.2 [64].

Results

Methodological results

The purpose of this first set of results is to validate our methodological approach and choices, they will not be the subject of discussion. No statistical asymmetry was observed in the funnel plot (z = -1.74; P = 0.08; S1~~2~~ Fig), a wide range of results and significance levels were represented by the studies included in this meta-analysis. The total heterogeneity among the effect sizes was statistically significant ($Q_{df=23}$ = 140.16; P <

0.0001), which indicated greater total heterogeneity than expected by the sampling error alone. The estimated amount of this total heterogeneity among the effect sizes was T^2 = 2.01; 95% confidence interval [CI] = 1.05-4.90. Of that total heterogeneity~~variability~~, a large amount (I^2 = 89.44%; CI = 81.59-95.39) was due to true heterogeneity between the studies, rather than just methodological heterogeneity~~variability~~. Thus, further examination of the true heterogeneity is warranted and was~~ill be~~ done through the analysis of multiple moderators.

Meta-analysis results

The meta-analysis of 24 studies (107 pairs of observations; S1 Table) suggests that hydropower dams and reservoirs did ~~not~~ have a statistically significant effect on macroinvertebrate richness. The mean effect size (*i.e.*, Hedge's *g*) estimate of our RVE model was -0.84 (95% CI = -1.62 to -0.05; P = 0.04), without accounting for the different moderators (Fig 3~~2~~). The large confidence interval not overlapping zero indicates that the mean effect size is statistically significant, but also highlights a wide range of effect sizes across studies. The sensitivity analysis shows that the estimates of the mean effect size and standard errors, as well as the estimate of between-study variance in study-average effect sizes (τ^2), are relatively insensitive to different value of ρ (S4 Table).

INSERT FIG 3~~2~~ HERE

Fig 3~~2~~. Forest plot of the meta-analysis~~.,~~ T~~t~~he mean effect size is -0.84 (95% CI = -1.62 to -0.05, shaded grey area), where study type is shape-coded (*i.e.*, circle for longitudinal studies and squares for cross-sectional studies) and biome color coded (*i.e.*, boreal in blue, temperate in yellow and tropical in red). A negative effect size means that there is a negative impact of hydropower in impacted sites as opposed to reference sites, whereas a positive effect size means that there is positive impact of hydropower in impacted sites as opposed to reference sites.

Moderators had very little influence in the effects of dams and reservoirs on macroinvertebrate richness. Biome did not significantly explain variability effects sizes. For this moderator, it was only possible to make statistical~~ly~~ interpretation ~~valid inferences~~

for the temperate level (estimate = -0.23; 95% CI = -1.97 to 1.51; P = 0.74; Fig 4~~3~~A) and tropical level (estimate = 0.02; 95% CI = -4.53 to 4.57; P = 0.99; Fig 4~~3~~A). We can interpret with statistical confidence that temperate and tropical biome did not significantly differ from zero. The boreal level had too few~~little~~ degrees of freedom (df_s < 4), ~~),~~ which invalidates the Satterthwaithe approximation (*i.e.*, ~~(*i.e.*,~~ calculation of the effective df_s of a linear combination of independent sample variances; [63,65,66]). ~~,~~Whether it is significant or not, we cannot interpret the boreal result with strong statistical confidence~~-~~. T~~t~~hus, the results of the RVE with this moderator in the equation~~s~~ have to be interpreted with caution. On the contrary, both type of impact and study moderators had enough df_s (for all levels) for robust statistical analysis. Statistically significant effects were neither found for downstream flow regulation (estimate = -0.50; 95% CI = -1.24 to 0.25; P = 0.18) nor for upstream water level fluctuations/drawdown impact types (estimate = -1.20; 95% CI = -3.45 to 1.05; P = 0.26; Fig 4~~3~~B). When study type was used as a moderator, there was no significant difference in effect sizes for cross-sectional design studies (*i.e.*, natural versus impacted; estimate = 0.45; 95% CI = -1.14 to 2.03; P = 0.56) and ~~, but there was a marginally significant difference for~~ longitudinal gradient type studies (*i.e.*, spatial gradient; estimate = -1.11; 95% CI = -2.32 to 0.10; P = 0.07; Fig 4~~3~~C). However, we can observe a visual trend where s~~S~~tudies that were considered as gradients were most often associated with negative effect sizes (not supported statistically). As for results from the season moderator, conclusions can only be drawn for the summer and winter levels. Statistically significant effects were neither found for summer (estimate = -0.46; 95% CI = -2.26 to 1.24; P = 0.58) nor for winter (estimate = -0.39; 95% CI = -2.83 to 2.05; P = 0.70; Fig 4~~3~~D). Results are similar for the sampling gear moderator, statistical inference can only

be drawn for the grab level, where no statistically significant effect can be observed (estimate = -0.67; 95% CI = -8.18 to 6.85; P = 0.83; Fig 4~~3~~E), other levels cannot be interpreted with confidence due to insufficient df_s (Satterthwaithe approximation invalidated).

INSERT FIG 4~~3~~ HERE

Fig 4~~3~~. Plots showing the mean effect sizes and their confidence interval for each of the moderators.~~.~~ Value in black is the mean effect size of the meta-analysis and the other colors are related to the different effect sizes when including specific moderators. When in grey, statistical significance of moderator cannot be interpreted with confidence due to statistical power issues (df_s insufficient). When effect size is in color (*i.e.*, blue) statistical interpretation can be made with confidence, whether it is significant or not (sufficient df_s). Asterisk signifies statistically marginally significant effect.

Discussion

The meta-analysis conducted on 107 pairs of observations (*i.e.*, impacted versus reference) extracted from 24 studies suggests that hydropower does statistically impact macroinvertebrate richness in a clear directional way. M~~Hydropower impacts m~~acroinvertebrates~~, their~~ richness in hydropower, dams and reservoirs impacted environments is significantly lower than in unimpacted environments (Fig. 2). To our knowledge, this meta-analysis is the first to resolve the result divergence observed in the existing literature. In fact, more than 88% of the effect sizes were significantly different from zero~~,~~ with ~~from which~~ 56% of the observations showing~~ed~~ reduced richness due to hydropower and 32% of the observations showing~~ed~~ reduced richness in reference conditions. These percentages, along with the statistically significant mean effect size (*i.e.*, -0.84; 95% CI = -1.62 to -0.05; P = 0.04), suggest a predominance of reduced macroinvertebrate richness due to hydropower, dams and reservoirs.

However, we also observed a large heterogeneity in macroinvertebrate richness responses to hydropower~~, which is most likely responsible for the marginality of the~~

statistical significance observed in the meta-analysis (Fig 32). Part of this variability across observations and studies can usually be explained by environmental and methodological variability, which we can try to control using moderators, and thus potentially strengthen the statistical significance of the result. In our meta-analysis, we considered biome (*i.e.*, boreal, temperate or tropical), type of impact (*i.e.*, water level fluctuations in the reservoir or flow regulation downstream of the dam), the type of study (*i.e.*, spatial longitudinal gradient [upstream vs downstream] or cross-sectional [natural lake versus impacted reservoir), sampling season (*i.e.*, spring, summer, fall and winter) and sampling gear (*i.e.*, net, grab and colonization basket) as moderators. Despite the documented effects on macroinvertebrate richness of the moderators included in our analysis [27,29,30,32,33], none of our moderators statistically explained heterogeneity in macroinvertebrate richness responses to hydropower.

Formatted: Highlight

Biome moderator

In general, it is quite common to observe a latitudinal gradient in species richness, where there is a maximum richness in the tropics and a decline towards the poles [30,31,67,68]. In a meta-analysis, Turgeon et al. [69], showed that the impacts of impoundments on fish richness was different across biomes. Significant declines in richness were observed in the tropics, a lower decline was observed in temperate regions and no impacts was observed in boreal biomes. Thus, we hypothesized that a latitudinal gradient could also influence the mean effect size in macroinvertebrates (*e.g.*, losing one species over a few species is more costly in terms of biodiversity loss, than over multiple species). Our data did not support a latitudinal trend which is not too surprising because there is no clear pattern about whether or not macroinvertebrate richness follows a

latitudinal gradient [70–72]. Pearson and Boyero [73] observed a richness peak around the equator for dragonflies (*i.e.*, odonata), but no clear global pattern for caddisflies (*i.e.*, trichoptera), whereas Vinson and Hawkins [71] observed richness peaks at mid-latitudes in South and North America for mayflies (*i.e.*, ephemeroptera), stoneflies (*i.e.*, plecoptera) and caddisflies (*i.e.*, trichoptera; [EPT] orders). On the contrary, Scott et al. [74] did not observe this latitudinal gradient for EPT in northern Canada. Geographic range (*i.e.*, narrow range and exclusion of extreme latitudes; [71]), sampling effort [75], macroinvertebrate specific life-history strategies [73,74] and data resolution (*i.e.*, weak gradient for local species richness; [76]) were probably the reason behind this lack of consensus [77], and absence of a trend in this meta-analysis. Moreover, the unbalanced sample size across biomes may have impeded any clear conclusions. Boreal samples represented 9% of the data, tropical samples 11% and roughly 79% of the data belong to temperate ecosystems. This prevented us from statistically supporting a trend for both boreal and tropical observations.

Impact type moderator

Water level fluctuation in reservoirs is characterized by yearly drawdown (*i.e.*, long-term oscillations in the water level), whereas flow regulation usually is reflected by daily or weekly changes in flow downstream of a dam (*i.e.*, short-term oscillations; [20]), keeping in mind that variation in the reservoir are also reflected downstream. In reservoir experiencing water level fluctuations in the form of winter drawdown (21% of the reservoirs in this meta-analysis; littoral zone samples), the littoral zone is exposed to desiccation and freezing for an extended period of time, which ~~has~~ caused ~~been causing~~ a loss of macroinvertebrate taxa [20,21,78,79] and decreased their overall abundance [80].

In the case of daily downstream fluctuations (79% of the reservoirs in this meta-analysis), organisms can burrow in the sediment (*i.e.*, low mobility taxa such as oligochaetes) or follow the water level up and down (*i.e.*, high mobility swimming taxa such as dragonfly nymphs; [20]). Riverine organisms have evolved and developed adaptations to survive flood and drought hydrological dynamics [81], but lentic taxa are not adapted to extreme water level fluctuations, such as winter drawdown in reservoirs (> 2m amplitude; [22]). Because of that, we were expecting a higher impact and effect size of water level fluctuation in reservoir (*i.e.*, winter drawdown) on macroinvertebrates richness than downstream flow regulation but found no difference nor significant effect of impact type moderator.

Study type moderator

Another moderator that could explain heterogeneity in our results was the type of study. An ideal way to analyze the impact of hydropower on richness is to set the reference conditions as richness before impoundment versus impacted conditions as richness after impoundment, within the same ecosystem (*e.g.*, a reference river that was impounded into an impacted reservoir; longitudinal in time, also known as BACI; [32]). However, studies using this type of methodology are rare, even more so for macroinvertebrates, and such studies were mostly absent in the results from the literature review and if present, did not fulfill the required criteria to be included in the meta-analysis ~~(none could be included in this case)~~. Here, we accounted for two types of study methodologies; a cross-sectional methodology (*i.e.*, comparing a hydropower impacted ecosystem with a natural/reference ecosystem; 33%) and longitudinal gradient in space methodology (*i.e.*, upstream of a dam versus downstream of that same dam, within a single ecosystem; 67%), which are both

considered as simple CI studies [32]. These methodologies are not ideal, compared to the longitudinal in time methodology (*i.e.*, BACI or BA studies), as their reference spatial point differs from the impacted spatial point, thus introducing some environmental noise and there is no way to control for environmental stochasticity [32]. In the cross-sectional type, studies included inter-ecosystem's variation (*i.e.*, impacted ecosystem was spatially independent from reference/non-impacted ecosystem). This inherently added some heterogeneity in the responses and the impacts could be more difficult to detect. In the longitudinal in space studies (reference upstream and impacted downstream of the dam, at a single point in time), the observations were from the same ecosystem. Here, there is less heterogeneity and thus, we could have expected the results to be less variable. However, there was no significant difference between the two study types in this meta-analysis. Even though the patterns were not significant, we observed a visual negative trend in the spatially longitudinal gradient studies (*i.e.*, higher richness in reference ecosystems, that is upstream of the dam), with a slightly tighter range of variation (not statistically supported). The cross-sectional studies had a tendency toward higher richness in reservoirs, with a larger range of variation. This might highlight a problem with general study design and the choice of the reference ecosystem, and caution is in order when interpreting these trends. Nevertheless, we believe that cross-sectional and longitudinal in space references are the best benchmark available to overcome current limitations regarding the lack of richness data before impoundment in the literature.

Sampling season and gear moderators

We, initially thought we would observe an effect of sampling season when comparing effect sizes since diversity and abundance is known to fluctuate yearly [33] but

there was no statistically significant effect of any season that could modulate the meta-analysis outcome. Similarly, we though that depending on the sampling gear we would observe varying diversity because different stratum would be sampled, thus representing different communities of macroinvertebrates [27] but no additional amount of heterogeneity was explained by the sampling gear moderator. The unbalanced nature of the sample sizes could be one of the reasons why these moderators do not provide statistically significant inferences.

The analysis of moderators did not allow a better understanding of the large heterogeneity in the effect sizes and suggests that maybe other moderators, which were not available for the studies included in this meta-analysis, could help tease out some of that heterogeneity. For instance, given that macroinvertebrate are very adapted to their localized environmental conditions (*e.g.*, temperature, granulometry, wave disturbance and macrophytes; [34]), their richness maybe regulated at a much finer scale. Thus, these micro-habitat moderators could be especially relevant to include in a future meta-analysis, although very hard to collect in such a global consolidating endeavour.

Conclusion

This meta-analysis suggested that there is a clear, directional, statistically significant conclusion regarding whether or not hydropower impacts macroinvertebrate richness; macroinvertebrate richness in hydropower, dams and reservoirs impacted environments is significantly lower than in unimpacted environments. However, we also observed a large range of effect sizes, from very negative to very positive impacts of hydropower. The environmental and methodological heterogeneity in the studies might have hindered the detection of a stronger significant effect, unfortunately none of our

moderators helped untangle that heterogeneity. This advises that other moderators, not included in this study due to unavailability among the studies, may be responsible for some of that heterogeneity. We advocate that local, smaller-scale variables pertaining to habitat physicochemical characteristics may bring some clarity about the large heterogeneity in effect sizes. As new studies evaluating the impacts of hydropower on macroinvertebrate richness accumulate, we would recommend that information regarding local habitat variables be available so they could be recorded and evaluated as moderators in future meta-analyses. This meta-analysis was able to highlight a clear directional effect of hydropower on macroinvertebrate richness. As richness is only one aspect of biodiversity, it would be interesting, in future studies, to conduct additional analyses of community composition and functional diversity and thus, get a better portrait of the impact of hydropower on macroinvertebrate biodiversity, not only richness. Moreover, as new studies are available,~~Nonetheless,~~ it would be~~ even more~~ interesting to keep enriching this meta-analysis~~it, as new studies are available,~~ to see if the results statistical confidence ~~we have in the results~~ could strengthen ~~in a few years~~ and become even more assertive.

Acknowledgements

We thank the CIRAIG – Polytechnique Montréal for covering the publication fees. We also thank the CSBQ for offering systematic reviews and meta-analyses workshops, which were more than useful for putting together this meta-analysis research article.

References

[1] Strayer DL, Dudgeon D. Freshwater biodiversity conservation: recent progress and future challenges. J North Am Benthol Soc 2010;29:344–58. https://doi.org/10.1899/08-171.1.
[2] Vörösmarty CJ, McIntyre PB, Gessner MO, Dudgeon D, Prusevich A, Green P, et al. Global threats to human water security and river biodiversity. Nature 2010;467:555–61. https://doi.org/10.1038/nature09440.
[3] Dudgeon D, Arthington AH, Gessner MO, Kawabata Z-I, Knowler DJ, Lévêque C, et al. Freshwater biodiversity: importance, threats, status and conservation challenges. Biol Rev 2006;81:163–82. https://doi.org/10.1017/S1464793105006950.
[4] Gracey EO, Verones F. Impacts from hydropower production on biodiversity in an LCA framework—review and recommendations. Int J Life Cycle Assess 2016;21:412–28. https://doi.org/10.1007/s11367-016-1039-3.
[5] Ward JV, Stanford JA. Ecological connectivity in alluvial river ecosystems and its disruption by flow regulation. Regul Rivers Res Manag 1995;11:105–19. https://doi.org/10.1002/rrr.3450110109.
[6] Kummu M, Varis O. Sediment-related impacts due to upstream reservoir trapping, the Lower Mekong River. Geomorphology 2007;85:275–93. https://doi.org/10.1016/j.geomorph.2006.03.024.
[7] Nilsson C, Brown RL, Jansson R, Merritt DM. The role of hydrochory in structuring riparian and wetland vegetation. Biol Rev 2010;85:837–58. https://doi.org/10.1111/j.1469-185X.2010.00129.x.
[8] Poff NL, Allan JD, Bain MB, Karr JR, Prestegaard KL, Richter BD, et al. The Natural Flow Regime. BioScience 1997;47:769–84. https://doi.org/10.2307/1313099.
[9] Bunn SE, Arthington AH. Basic Principles and Ecological Consequences of Altered Flow Regimes for Aquatic Biodiversity. Environ Manage 2002;30:492–507. https://doi.org/10.1007/s00267-002-2737-0.
[10] Friedl G, Wüest A. Disrupting biogeochemical cycles - Consequences of damming. Aquat Sci 2002;64:55–65. https://doi.org/10.1007/s00027-002-8054-0.
[11] Santucci VJ, Gephard SR, Pescitelli SM. Effects of Multiple Low-Head Dams on Fish, Macroinvertebrates, Habitat, and Water Quality in the Fox River, Illinois. North Am J Fish Manag 2005;25:975–92. https://doi.org/10.1577/M03-216.1.
[12] Poff NL, Olden JD, Merritt DM, Pepin DM. Homogenization of regional river dynamics by dams and global biodiversity implications. Proc Natl Acad Sci 2007;104:5732–7. https://doi.org/10.1073/pnas.0609812104.
[13] Liermann CR, Nilsson C, Robertson J, Ng RY. Implications of Dam Obstruction for Global Freshwater Fish Diversity. BioScience 2012;62:539–48. https://doi.org/10.1525/bio.2012.62.6.5.
[14] Jackson HM, Gibbins CN, Soulsby C. Role of discharge and temperature variation in determining invertebrate community structure in a regulated river. River Res Appl 2007;23:651–69. https://doi.org/10.1002/rra.1006.
[15] Takao A, Kawaguchi Y, Minagawa T, Kayaba Y, Morimoto Y. The relationships between benthic macroinvertebrates and biotic and abiotic environmental characteristics downstream of the Yahagi Dam, Central Japan, and the State Change Caused by inflow

from a Tributary. River Res Appl 2008;24:580–97. https://doi.org/10.1002/rra.1135.

[16] Kullasoot S, Intrarasattayapong P, Phalaraksh C. Use of benthic macroinvertebrates as bioindicators of anthropogenic impacts on water quality of Mae Klong river, Western Thailand. Chiang Mai J Sci 2017;44:1356–66.

[17] Kraft KJ. Effect of Increased Winter Drawdown on Benthic Macroinvertebrates in Namakan Reservoir, Voyeurs National Park. Houghton, MI, USA: Michigan Techonological University; 1988.

[18] Englund G, Malmqvist B. Effects of Flow Regulation, Habitat Area and Isolation on the Macroinvertebrate Fauna of Rapids in North Swedish Rivers. Regul Rivers Res Manag 1996;12:433–45. https://doi.org/10.1002/(SICI)1099-1646(199607)12:4/5<433::AID-RRR415>3.0.CO;2-6.

[19] Malmqvist B, Englund G. Effects of hydropower-induced flow perturbations on mayfly (Ephemeroptera) richness and abundance in north Swedish river rapids. Hydrobiologia 1996;341:145–58. https://doi.org/10.1007/BF00018118.

[20] Valdovinos C, Moya C, Olmos V, Parra O, Karrasch B, Buettner O. The importance of water-level fluctuation for the conservation of shallow water benthic macroinvertebrates: an example in the Andean zone of Chile. Biodivers Conserv 2007;16:3095–109. https://doi.org/10.1007/s10531-007-9165-7.

[21] Aroviita J, Hämäläinen H. The impact of water-level regulation on littoral macroinvertebrate assemblages in boreal lakes. Hydrobiologia 2008;613:45–56. https://doi.org/10.1007/s10750-008-9471-4.

[22] White MS, Xenopoulos MA, Metcalfe RA, Somers KM. Water level thresholds of benthic macroinvertebrate richness, structure, and function of boreal lake stony littoral habitats. Can J Fish Aquat Sci 2011;68:1695–704. https://doi.org/10.1139/f2011-094.

[23] Głowacki Ł, Grzybkowska M, Dukowska M, Penczak T. Effects of damming a large lowland river on chironomids and fish assessed with the (multiplicative partitioning of) true/Hill biodiversity measure. River Res Appl 2011;27:612–29. https://doi.org/10.1002/rra.1380.

[24] Serafini Floss EC, Secretti E, Kotzian CB, Spies MR, Pires MM. Spatial and temporal distribution of non-biting midge larvae assemblages in streams in a mountainous region in southern Brazil. J INSECT Sci 2013;13.

[25] Smokorowski KE, Metcalfe RA, Finucan SD, Jones N, Marty J, Power M, et al. Ecosystem level assessment of environmentally based flow restrictions for maintaining ecosystem integrity: a comparison of a modified peaking versus unaltered river. Ecohydrology 2011;4:791–806. https://doi.org/10.1002/eco.167.

[26] Marchetti MP, Esteban E, Smith ANH, Pickard D, Richards AB, Slusark J. Measuring the ecological impact of long-term flow disturbance on the macroinvertebrate community in a large Mediterranean climate river. J Freshw Ecol 2011;26:459–80. https://doi.org/10.1080/02705060.2011.577974.

[27] De Palma A, Sanchez-Ortiz K, Martin PA, Chadwick A, Gilbert G, Bates AE, et al. Chapter Four - Challenges With Inferring How Land-Use Affects Terrestrial Biodiversity: Study Design, Time, Space and Synthesis. In: Bohan DA, Dumbrell AJ, Woodward G, Jackson M, editors. Adv. Ecol. Res., vol. 58, Academic Press; 2018, p. 163–99. https://doi.org/10.1016/bs.aecr.2017.12.004.

[28] Viechtbauer W. Conducting Meta-Analyses in with the metafor Package. J Stat Softw 2010;36:1–48. https://doi.org/10.18637/jss.v036.i03.

[29] Gibson L, Lee TM, Koh LP, Brook BW, Gardner TA, Barlow J, et al. Primary forests are irreplaceable for sustaining tropical biodiversity. Nature 2011;478:378–81. https://doi.org/10.1038/nature10425.
[30] Willig MR, Kaufman DM, Stevens RD. Latitudinal Gradients of Biodiversity: Pattern, Process, Scale, and Synthesis. Annu Rev Ecol Evol Syst 2003;34:273–309. https://doi.org/10.1146/annurev.ecolsys.34.012103.144032.
[31] Rosenzweig ML. Species diversity in space and time. Cambridge, United Kingdom: Cambridge University Press; 1995.
[32] Christie AP, Amano T, Martin PA, Shackelford GE, Simmons BI, Sutherland WJ. Simple study designs in ecology produce inaccurate estimates of biodiversity responses. J Appl Ecol 2019;56:2742–54. https://doi.org/10.1111/1365-2664.13499.
[33] Hill MJ, Sayer CD, Wood PJ. When is the best time to sample aquatic macroinvertebrates in ponds for biodiversity assessment? Environ Monit Assess 2016;188:188–94. https://doi.org/10.1007/s10661-016-5178-6.
[34] McCafferty WP. Aquatic Entomology: The Fishermen's and Ecologists' Illustrated Guide to Insects and Their Relatives. Sudbury, MA, USA: Jones and Bartlett Publishing; 1983.
[35] Wetzel RG. Limnology: Lake and river ecosystems. Third Edition. San Diego, CA, USA: Academic Press; 2001.
[36] Koricheva J, Gurevitch J, Mengersen K, editors. Handbook of Meta-Analysis in Ecology and Evolution. Princeton, NJ, USA: Princeton University Press; 2013.
[37] Moher D. Preferred Reporting Items for Systematic Reviews and Meta-Analyses: The PRISMA Statement. Ann Intern Med 2009;151:264. https://doi.org/10.7326/0003-4819-151-4-200908180-00135.
[38] Clarivate Analytics. Browse, search, and explore journals indexed in the Web of Science. Web Sci Group Clarivate Anal Co 2019. https://mjl.clarivate.com/ (accessed November 27, 2019).
[39] Aroviita J, Hämäläinen H. The impact of water-level regulation on littoral macroinvertebrate assemblages in boreal lakes. Hydrobiologia 2008;613:45–56. https://doi.org/10.1007/s10750-008-9471-4.
[40] Molozzi J, Hepp LU, Callisto M. The additive partitioning of macroinvertebrate diversity in tropical reservoirs. Mar Freshw Res 2013;64:609–17. https://doi.org/10.1071/MF12354.
[41] Mellado-Diaz A, Sanchez-Gonzalez JR, Guareschi S, Magdaleno F, Velasco MT. Exploring longitudinal trends and recovery gradients in macroinvertebrate communities and biomonitoring tools along regulated rivers. Sci Total Environ 2019;695:133774. https://doi.org/10.1016/j.scitotenv.2019.133774.
[42] Bruno D, Belmar O, Maire A, Morel A, Dumont B, Datry T. Structural and functional responses of invertebrate communities to climate change and flow regulation in alpine catchments. Glob Change Biol 2019;25:1612–28. https://doi.org/10.1111/gcb.14581.
[43] Milner VS, Yarnell SM, Peek RA. The ecological importance of unregulated tributaries to macroinvertebrate diversity and community composition in a regulated river. Hydrobiologia 2019;829:291–305. https://doi.org/10.1007/s10750-018-3840-4.
[44] Steel AE, Peek RA, Lusardi RA, Yarnell SM. Associating metrics of hydrologic variability with benthic macroinvertebrate communities in regulated and unregulated

snowmelt-dominated rivers. Freshw Biol 2018;63:844–58. https://doi.org/10.1111/fwb.12994.

[45] Schneider SC, Petrin Z. Effects of flow regime on benthic algae and macroinvertebrates - A comparison between regulated and unregulated rivers. Sci Total Environ 2017;579:1059–72. https://doi.org/10.1016/j.scitotenv.2016.11.060.

[46] Vaikasas S, Palaima K, Pliūraite V. Influence of hydropower dams on the state of macroinvertebrates assemblages in the Virvyte river, Lithuania. J Environ Eng Landsc Manag 2013;21:305–15. https://doi.org/10.3846/16486897.2013.796956.

[47] Dolédec S, Simon L, Blemus J, Rigal A, Robin J, Mermillod-Blondin F. Multiple stressors shape invertebrate assemblages and reduce their trophic niche: A case study in a regulated stream. Sci Total Environ 2021;773:145061. https://doi.org/10.1016/j.scitotenv.2021.145061.

[48] Wang H, Chen Y, Liu Z, Zhu D. Effects of the “Run-of-River” Hydro Scheme on Macroinvertebrate Communities and Habitat Conditions in a Mountain River of Northeastern China. Water 2016;8:31. https://doi.org/10.3390/w8010031.

[49] Nukazawa K, Shirasaka K, Kajiwara S, Saito T, Irie M, Suzuki Y. Gradients of flow regulation shape community structures of stream fishes and insects within a catchment subject to typhoon events. Sci Total Environ 2020;748:141398. https://doi.org/10.1016/j.scitotenv.2020.141398.

[50] Quadroni S, Salmaso F, Gentili G, Crosa G, Espa P. Response of benthic macroinvertebrates to different hydropower off-stream diversion schemes. Ecohydrology 2020;n/a:e2267. https://doi.org/10.1002/eco.2267.

[51] Vilenica M, Pozojević I, Vučković N, Mihaljević Z. How suitable are man-made water bodies as habitats for Odonata? Knowl Manag Aquat Ecosyst 2020:13. https://doi.org/10.1051/kmae/2020008.

[52] Uieda VS, Marçal SF. How the structure if a phytophilous chironomid assemblage responds to a lake level drawdown for submerged macropohyte control in a tropical reservoir. Limnetica 2020;39:555–69.

[53] Cazaubon A, Giudicelli J. Impact of the residual flow on the physical characteristics and benthic community (algae, invertebrates) of a regulated Mediterranean river: the Durance, France. Regul Rivers Res Manag 1999;15:441–61. https://doi.org/10.1002/(SICI)1099-1646(199909/10)15:5<441::AID-RRR558>3.0.CO;2-9.

[54] Olson DM, Dinerstein E, Wikramanayake ED, Burgess ND, Powell GVN, Underwood EC, et al. Terrestrial Ecoregions of the World: A New Map of Life on EarthA new global map of terrestrial ecoregions provides an innovative tool for conserving biodiversity. BioScience 2001;51:933–8. https://doi.org/10.1641/0006-3568(2001)051[0933:TEOTWA]2.0.CO;2.

[55] Mitchell M, Muftakhidinov B, Winchen T. Engauge Digitizer - Extract data points from images of graphs 2017. http://markummitchell.github.io/engauge-digitizer (accessed March 3, 2012).

[56] Borenstein M, Hedges LV, Higgins JPT, Rothstein HR. Introduction to Meta-Analysis. United Kingdom: John Wiley & Sons; 2011.

[57] Hedges LV. Distribution Theory for Glass’s Estimator of Effect size and Related Estimators. J Educ Stat 1981;6:107–28. https://doi.org/10.3102/10769986006002107.

[58] Wickham H. ggplot2: Elegant Graphics for Data Analysis. Springer-Verlag New

York; 2016.
[59] Sterne JAC, Egger M. Funnel plots for detecting bias in meta-analysis: Guidelines on choice of axis. J Clin Epidemiol 2001;54:1046–55. https://doi.org/10.1016/S0895-4356(01)00377-8.
[60] Sterne JAC, Egger M. Regression methods to detect publication and other bias in meta-analysis. In: Rothstein HR, Sutton AJ, Borenstein M, editors. Publ. Bias Meta-Anal. Prev. Assess. Adjust. First Edition, Chichester, United Kingdom: Wiley; 2005, p. 99–110.
[61] Huedo-Medina TB, Sánchez-Meca J, Marín-Martínez F, Botella J. Assessing heterogeneity in meta-analysis: Q statistic or I^2 index? Psychol Methods 2006;11:193–206. https://doi.org/10.1037/1082-989X.11.2.193.
[62] Moeyaert M, Ugille M, Beretvas SN, Ferron J, Bunuan R, Noortgate WV den. Methods for dealing with multiple outcomes in meta-analysis: a comparison between averaging effect sizes, robust variance estimation and multilevel meta-analysis. Int J Soc Res Methodol 2017;20:559–72. https://doi.org/10.1080/13645579.2016.1252189.
[63] Fisher Z, Tipton E. robumeta: an R package for robust variance estimation in meta-analysis 2017.
[64] R Core Team. R: a language and environment for statistical computing. Vienna, Austria: R Foundation for Statistical Computing; 2021.
[65] van Emden HF. Statistics for Terrified Biologists. First Edition. Oxford, United Kingdom: Wiley-Blackwell; 2008.
[66] Spellman FR, Whiting NE. Handbook of Mathematics and Statistics for the Environment. First Edition. Boca Raton, FL, USA: CRC Press; 2013.
[67] Hillebrand H. On the Generality of the Latitudinal Diversity Gradient. Am Nat 2004;163:192–211. https://doi.org/10.1086/381004.
[68] Lomolino MV, Riddle BR, Brown JH. Biogeography. 3rd ed. Sunderland, MA, USA: Sinauer Associates; 2006.
[69] Turgeon K, Turpin C, Gregory- Eaves I. Dams have varying impacts on fish communities across latitudes: a quantitative synthesis. Ecol Lett 2019;22:1501–16. https://doi.org/10.1111/ele.13283.
[70] Covich AP. Geographical and Historical Comparisons of Neotropical Streams: Biotic Diversity and Detrital Processing in Highly Variable Habitats. J North Am Benthol Soc 1988;7:361–86. https://doi.org/10.2307/1467297.
[71] Vinson MR, Hawkins CP. Broad-scale geographical patterns in local stream insect genera richness. Ecography 2003;26:751–67. https://doi.org/10.1111/j.0906-7590.2003.03397.x.
[72] Allan JD, Castillo MM. Stream Ecology: Structure and Function of Running Waters. Second Edition. The Netherlands: Springer Science & Business Media; 2007.
[73] Pearson RG, Boyero L. Gradients in regional diversity of freshwater taxa. J North Am Benthol Soc 2009;28:504–14. https://doi.org/10.1899/08-118.1.
[74] Scott RW, Barton DR, Evans MS, Keating JJ. Latitudinal gradients and local control of aquatic insect richness in a large river system in northern Canada. J North Am Benthol Soc 2011;30:621–34. https://doi.org/10.1899/10-112.1.
[75] Vinson MR, Hawkins CP. Effects of Sampling Area and Subsampling Procedure on Comparisons of Taxa Richness among Streams. J North Am Benthol Soc 1996;15:392–9. https://doi.org/10.2307/1467286.
[76] Heino J. Biodiversity of Aquatic Insects: Spatial Gradients and Environmental

Correlates of Assemblage-Level Measures at Large Scales. Freshw Rev 2009;2:1–29. https://doi.org/10.1608/FRJ-2.1.1.

[77] Shah DN, Domisch S, Pauls SU, Haase P, Jähnig SC. Current and future latitudinal gradients in stream macroinvertebrate richness across North America. Freshw Sci 2014;33:1136–47. https://doi.org/10.1086/678492.

[78] Palomäki R, Koskenniemi E. Effects of bottom freezing on macrozoobenthos in the regulated lake Pyhäjärvi. Arch Für Hydrobiol 1993;128:73–90.

[79] Palomäki R. Response by macrozoobenthos biomass to water level regulation in some Finnish lake littoral zones. Hydrobiologia 1994;286:17–26. https://doi.org/10.1007/BF00007277.

[80] Trottier G, Embke H, Turgeon K, Solomon C, Nozais C, Gregory-Eaves I. Macroinvertebrate abundance is lower in temperate reservoirs with higher winter drawdown. Hydrobiologia 2019;834:199–211. https://doi.org/10.1007/s10750-019-3922-y.

[81] Humphries P, Baldwin DS. Drought and aquatic ecosystems: an introduction. Freshw Biol 2003;48:1141–6. https://doi.org/10.1046/j.1365-2427.2003.01092.x.

Supporting information

~~**S1 Fig.** PRISMA flow diagram for this meta-analysis from Moher et al. [37].~~

S1 Table. PRISMA checklist. Specific to~~for~~ this meta-analysis, from Moher et al. [37].

S1~~2~~ Fig. Funnel plot for this meta-analysis.~~,~~ N~~where n~~o statistically significant asymmetry is observed (z = -1.74, P = 0.08).

S2 Table. Metadata table showing all variables for each study~~ies~~ included in this meta-analysis [14-18, 20, 22, 25-26, 40-54]. Use S3 Table as a companion table to get more information on each variable.

S3 Table. Companion table describing all variables in S2 Table.

S4 Table. Table showing the sensitive analysis outputs. Rho values (ρ) ranges from 0 to 1 and mean effect size (ES), standard error (SE) and between study variance (τ^2) estimates are relatively insensitive to these varying ρ values.

Reviewers' comments:

Reviewer's Responses to Questions

Comments to the Author

1. Is the manuscript technically sound, and do the data support the conclusions?

The manuscript must describe a technically sound piece of scientific research with data that supports the conclusions. Experiments must have been conducted rigorously, with appropriate controls, replication, and sample sizes. The conclusions must be drawn appropriately based on the data presented.

Reviewer #1: Yes

Reviewer #2: Yes

2. Has the statistical analysis been performed appropriately and rigorously?

Reviewer #1: Yes

Reviewer #2: Yes

3. Have the authors made all data underlying the findings in their manuscript fully available?

The PLOS Data policy requires authors to make all data underlying the findings described in their manuscript fully available without restriction, with rare exception (please refer to the Data Availability Statement in the manuscript PDF file). The data should be provided as part of the manuscript or its supporting information, or deposited to a public repository. For example, in addition to summary statistics, the data points behind means, medians and variance measures should be available. If there are restrictions on publicly sharing data—e.g. participant privacy or use of data from a third party—those must be specified.

Reviewer #1: Yes

Reviewer #2: Yes

4. Is the manuscript presented in an intelligible fashion and written in standard English?

PLOS ONE does not copyedit accepted manuscripts, so the language in submitted articles must be clear, correct, and unambiguous. Any typographical or grammatical errors should be corrected at revision, so please note any specific errors here.

Reviewer #1: Yes

Reviewer #2: Yes

5. Review Comments to the Author

Please use the space provided to explain your answers to the questions above. You may also include additional comments for the author, including concerns about dual publication, research ethics, or publication ethics. (Please upload your review as an attachment if it exceeds 20,000 characters)

REVIEWER #1

Review of Trottier et al.
The authors of this manuscript, "The impacts of hydropower on freshwater macroinvertebrate richness: a global meta-analysis" assess the impacts of hydropower dams on macroinvertebrate richness. They conduct a meta-analysis of the literature, and attempt to account for several potential moderators, including the biome the studies occurred in; whether the study sites occurred upstream (within the reservoir) or downstream of the dam; the study design; the sampling season; and the type of sampling gear used. Using a random and mixed effect modelling approach, the authors conclude that hydropower does significantly impact macroinvertebrate richness, but they observed a large range of effect sizes that were not accounted for by the included moderators, and suggest including local habitat variables in future studies would be helpful in understanding the response of macroinvertebrate richness to hydropower impacts.

Overall, I thought the meta-analysis was well laid out and clear (following the PRISMA approach), however, there were a few areas of the manuscript that were confusing and require clarification. The authors focus their meta-analysis specifically on macroinvertebrate richness. In their introduction, they mention three aspects of biodiversity: richness, community assemblage and functional diversity. It might be helpful to provide some additional rationale as to why the decision to focus specifically on richness. Is richness more easily quantifiable and extractable for a meta-analyses? While functional diversity measures are likely more limited in the literature, community composition data is frequently assessed, and might also be insightful in looking at hydropower impacts. This might be beyond the scope of this paper, but perhaps some additional rationale to focusing on richness may be helpful.

*** Response: As it was pointed out by the reviewer, we focused this meta-analysis on macroinvertebrate richness because it was more easily quantifiable/extractable in this context. It took a lot of effort to access simple taxon richness data for 24 studies. As community composition and functional diversity are more data hungry and a lot more limited in term of accessibility and computability than taxon richness, they were not as easy to retrieve at the meta-analysis' scale. We added a statement at the end of the introduction (lines 134-137), as well as a sentence saying it would be interesting to conduct further studies using these biodiversity aspects in the conclusion (lines 601-604).

The authors included sampling gear as a proxy for habitat stratum (i.e. benthic grabs vs nets). This approach might need a caveat, as many biomonitoring protocols (including the EPA in the US) use nets for multiple-habitat assessments, whereas grabs are primarily used in deeper reservoir studies (upstream).

*** Response: We thank the reviewer for bringing this issue to our attention. We added a caveat inspired by the reviewer's suggestion (lines 115-119).

The authors included a comprehensive literature search using the Web of Science core collection, and provide the terms used. In the research strategy (line 208; Figure1), the authors state that they reduced the number of included papers to 24, but in the data collection section (Line 220) mention collecting metrics from 17 studies. There does not seem to be any mention on how the number of studies was reduced from 24 (Table S2 does list 24 studies).

*** Response: This was a typographical error. It was corrected to 24 studies.

The seasonal sampling moderator (spring/summer/fall/winter) seems very coarse. For either upstream draw down or downstream modifications in stream flow, were samples collected immediately after alterations or later in the season? As some macroinvertebrates are relatively mobile, they could recolonize shortly after short-term water regulation effects.

*** Response: This is true. However, since this is a meta-analysis, it was not easy to choose an appropriate coarseness for this specific moderator, especially considering some of the studies were not very specific about their sampling. We could have used Julian day, but we were more interested in the coarser temporal scale effect of seasons rather than micro changes in colonization following immediate water regulation. We have added justification to this choice in the manuscript (lines 131-133).

In the data collection/extraction section, it might be helpful to include information on whether multiple authors were involved in abstracting the data, or if this was done by a single analyst.

*** Response: We added this information to the data collection paragraph (lines 220-221).

For samples from reservoirs, it might be useful to know whether samples were collected in the center of the lake or in the littoral regions. The authors state (Line 518) they were expecting a large effect of water level fluctuations. If these were samples collected from the profundal region in the center of the lake, water level drawdown might not have a large impact.

*** Response: After verification, all samples from reservoir studies were collected in the littoral zone, not in the profundal zone. We specified types of samples at line 518.

Minor edits:

Line 477: The authors state “despite the documented effects on macroinvertebrate richness.” while these are discussed briefly elsewhere in the manuscript, It would be helpful to include some citations with this statement.

*** Response: We added appropriate references for this statement.

Line 496: states that the authors did not have a statistical supporting trend for both boreal and tropical, but previously (lines 395-397), they state they did make statistically valid inferences for both temperate and tropical biomes (just not for boreal).

*** Response: We could indeed make valid statistical inferences, but for both temperate and tropical biome, the confidence interval encompassed zero, so we can say with confidence that there is no statistically significant effect of tropical and temperate biomes, whereas for the boreal biome, we cannot even interpret with confidence the statistical trend observed. We added clarifications to minimize reader confusion (lines 395-398)

Overall, I thought the meta-analysis was interesting, perhaps it might be worth mentioning that in the discussing/conclusion that additional analyses of community composition and functional diversity would also be an important future direction in assessing the impacts of hydropower on macroinvertebrate communities. As mentioned by the authors in their introduction, taxon richness is only one aspect of biodiversity.

*** Response: We thank the reviewer for its valuable feedback. We added a sentence mentioning the future relevance of additional community composition and functional diversity analyses (lines 601-604).

REVIEWER #2

This paper describes a meta-analysis of the literature asking whether hydropower, dams, and reservoirs affect macroinvertebrate species richness. The authors found that a preponderance of studies found negative effects on species richness. However, there was a range of effect sizes in their dataset and despite having a number of likely moderators that might explain variation between studies, the authors found that these moderators were not effective in explaining effect size variation.

Generally this study was well conceived and completed. I liked the detailed description of how the meta-analysis was done and that they used the PRISMA methodology for conducing a meta-analysis.

One thing I’d like to see clarified is what is being asked of the moderators. Are you asking if the moderators impact species richness, or if the moderators explain heterogeneity in the effect sizes? I think it is the latter, but this could be made more clear. I suppose that’s why you call them moderators and not covariates?

*** Response: We are indeed asking the moderators to explain additional heterogeneity, as suggested by the reviewer. We added a sentence to clarify this matter in the manuscript (lines 312-314).

Although I felt there was a lack of detail explaining this aspect of the study, there are at times places where it felt like there was too much detail (see specific comments below).

In addition, a careful screening of the paper would be useful to address various grammatical mistakes – I've highlighted some below – which will make the paper more easily assessable to the reader.

Minor comments:

Line 30 and throughout (e.g., 292, 302, 303, 305): mixed effects models are mixed because they contain fixed and random effects. It is therefore redundant to say "random and mixed effect model".

*** Response: We thank the reviewer for bringing this matter to our attention. We reviewed this formulation and felt comfortable using "random/mixed-effects model" (as it is used in Viechtbauer [2010]) instead of "random and mixed effect model" throughout the manuscript.

Line 33-34: change "statistical way" to "statistically significant way".

*** Response: We changed it according to the reviewer suggestion.

Line 55: add comma before "but"

*** Response: We added a comma.

Line 72: between and "natural-like" flows?

*** Response: We added "impacted flows" (it became missing during the back and forth between co-authors revision of the manuscript pre-submission).

Line 74: delete "allow to"

*** Response: We thank the reviewer for taking the time to highlight these grammatical/syntax errors, we deleted "allow to".

Line 83-84: Why would this latitudinal effect on species richness affect effect sizes? Perhaps more likely to see effects in tropics than higher latitudes because a floor effect makes it difficult to detect change in richness? In any case, it would help the reader to explain the reasoning rather than leaving the reader to connect the dots.

*** Response: We are not entirely sure what the reviewer meant by “floor effect”. Nonetheless, we were able to clarify our reasoning and added few lines at two locations in the manuscript (lines 85-87 and 488-496) stating that the loss of one taxon over few taxa is relatively more important than over multiple taxa and thus, why we could potentially observe an effect of a latitudinal gradient on our effect size (*i.e.*, species richness highest in the tropics and lowest near the poles).

Lines 99-104: Most of these study designs are not in your dataset – or even likely as you point out. What isn’t mentioned here are the two study designs you did use in your models.

*** Response: The study designs found in our meta-analysis are in facts two variants of the Control-Impact sampling design (line 128-131), that is cross-sectional (*i.e.*, reference natural lake versus impacted reservoir) and longitudinal spatial gradient (*i.e.*, upstream of a dam [reservoir] versus downstream of a dam [river]). In lines 99-104, we simply make our case that sampling, or study design could affect the conclusions of a study.

Lines 137: A little overly wordy – does meta-analysis really need this much defense as a method for the audience of PLoS One?

*** Response: We deleted this paragraph. We agree meta-analysis does not need as much defense and description in PLOS ONE paper.

Line 183: Why this date (1989)?

*** Response: This is the earliest year you can search for articles in Web of Science, we specified it in the manuscript.

Line 198: Why was sorting required? If you went through every result, then sorting is irrelevant.

*** Response: We thank the reviewer for pointing that out. We deleted this part of the sentence.

Line 198: Change “studies” to “study”

*** Response: We changed “studies” to “study”.

Line 220: Everywhere else in the manuscript you say “24” studies.

*** Response: This was a typographical error. It has been corrected to 24.

Lines 287-290: This belongs in the next section, not here.

*** Response: We thank the reviewer for its feedback on the matter. However, without further justifications as to why this belong in the next section (next paragraph or in the

results?), we feel confident that it does belong where it is now and would prefer that it remains that way.

Lines 357-359: You call a “P = 0.07” “marginally significant” below.

*** Response: We rectified our statement regarding the p-value of 0.07 stating it was not statistically significant, but that visual assessment could detect a potential trend where studies that were considered as gradients were most often associated with negative effect sizes (not supported statistically; lines 408-413).

Lines 362-365: How is the “true heterogeneity” compared to the “methodological variability” assessed? Is this left-over variance not explained by your model?

*** Response: The methodological variability is the heterogeneity due to sampling error within each individual study included in the meta-analysis, whereas the true heterogeneity is due to specific study characteristics, which can introduce heterogeneity among the effect sizes. The combination of this methodological and true heterogeneity is referred to as total heterogeneity. We tried to clear that up in the methodology section (lines 279-290) so that lines 362-365 are easier to understand.

Line 366: Change “will be” to “was”.

*** Response: We changed “will be” to “was”.

Lines 369: You mean hydropower, dams, and reservoirs DID have a statistically significant effect on macroinvertebrate richness, right?

*** Response: Yes, this has been rectified.

Line 399: Change “little” to “few”.

*** Response: We changed “will be” to “was”.

Line 404: add “for all levels” after “df”

*** Response: We added “(for all levels)” after “df”.

Lines 448-450: Sentence begins in a way redundant with previous sentence. Change “Hydropower impacts macroinvertebrates, their richness…” to “Macroinvertebrate richness…”

*** Response: This has been rectified.

Line 452: Change “from which” to “with”

*** Response: We modified “from which” to “with” as well as “showed” to “showing” in the remainder of the sentence.

Lines 467: Why are you now calling this a marginal effect?

*** Response: This is a leftover typographical error from a previous version of this manuscript, we thank the reviewer for pointing it out. We deleted part of the sentence mentioning the “marginality” of the mean effect size which is not valid for the updated/enhanced meta-analysis (17 to 24 studies included).

Lines 472: Delete “and thus potentially strengthen the statistical significance of the result”. Awkward phrasing that doesn’t seem necessary.

*** Response: We thank the reviewer for its output, we deleted this part of the sentence.

Line 516: Change “been causing” to “caused”.

*** Response: We changed “been causing” to “caused”.

Lines 539: Delete “(none could be included in this case)”. Redundant.

*** Response: We deleted this parenthesis.

6. PLOS authors have the option to publish the peer review history of their article (what does this mean?). If published, this will include your full peer review and any attached files.

If you choose “no”, your identity will remain anonymous but your review may still be made public.

Do you want your identity to be public for this peer review? For information about this choice, including consent withdrawal, please see our Privacy Policy.

Reviewer #1: No

Reviewer #2: No
